# Supplementary material for: Interleukin-11 causes alveolar type 2 cell dysfunction and prevents alveolar regeneration
Source: Nat Commun. 2024 Oct 2;15:8530. doi: 10.1038/s41467-024-52810-8 (PMC11448503; doi:10.1038/s41467-024-52810-8)
Supplement: Supplementary file 1 — Supplementary Information [file 41467_2024_52810_MOESM1_ESM.pdf]

Interleukin-11 causes alveolar type 2 cell dysfunction and prevents alveolar regeneration

Authors

Benjamin Ng[1,2*], Kevin Y. Huang[2,3#], Chee Jian Pua[1#], Sivakumar Viswanathan[2], Wei-Wen Lim[1,2], Fathima F. Kuthubudeen[2], Yu-Ning Liu[2], An An Hii[1], Benjamin L. George[2], Anissa A. Widjaja[2], Enrico Petretto[2,3] and Stuart A. Cook[1,2,4*]

Affiliations

[1] National Heart Research Institute Singapore, National Heart Center Singapore, Singapore.

[2] Cardiovascular and Metabolic Disorders Program, Duke-National University of Singapore Medical School, Singapore.

[3] Center for Computational Biology, Duke-National University of Singapore Medical School, Singapore.

[4] MRC-London Institute of Medical Sciences, Hammersmith Hospital Campus, London, United Kingdom.

Corresponding authors. Email: benjamin.ng.w.m@nhcs.com.sg (B.N.); stuart.cook@duke-nus.edu.sg (S.A.C.)

This supplementary file contains:

Supplementary Figure 1-24

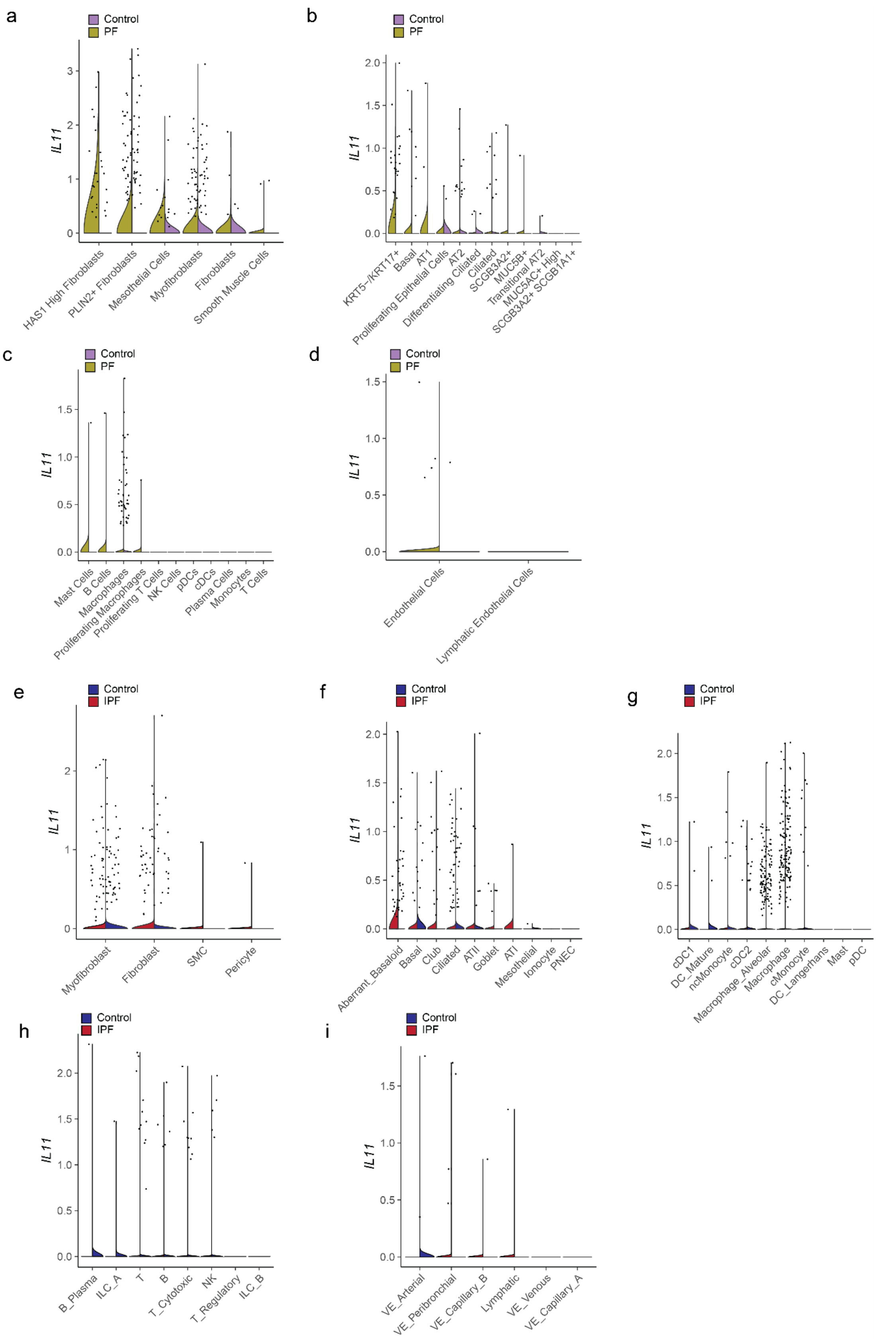

Supplementary Figure 1. ***IL11*** **is elevated across mesenchymal and epithelial cell subsets in human pulmonary fibrosis.** Violin plot of IL11 expression in individual cell types in scRNA-seq data from control and PF samples in (**a-d**) Habermann et. al. (GSE135893) and (**e-i**) Adams et. al. (GSE136831) datasets. Data are further grouped by PF or control in mesenchymal, epithelial, immune (myeloid or lymphoid) or endothelial cell types.

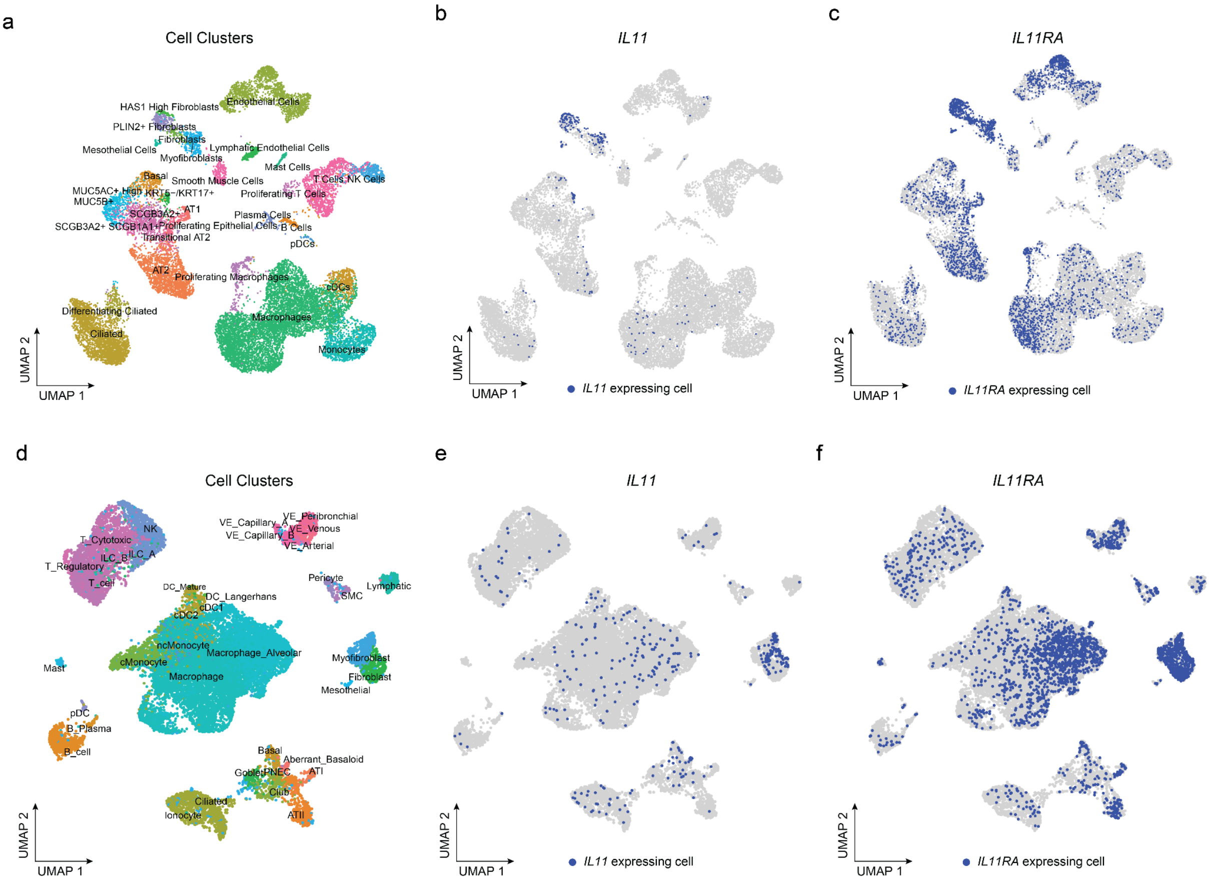

Supplementary Figure 2. UMAP visualization of *IL11* and *IL11RA*-expressing cells in the human lung. UMAP visualization of *IL11* or *IL11RA* expressing single cells in scRNA-seq data from control and PF samples in the (**a-c**) Habermann et. al. (GSE135893) and (**d-f**) Adams et. al. (GSE136831) dataset. Colored dots indicate different cell clusters in a and c. *IL11*- or *IL11RA*-expressing cells are colored in dark blue in b,c,e,f.

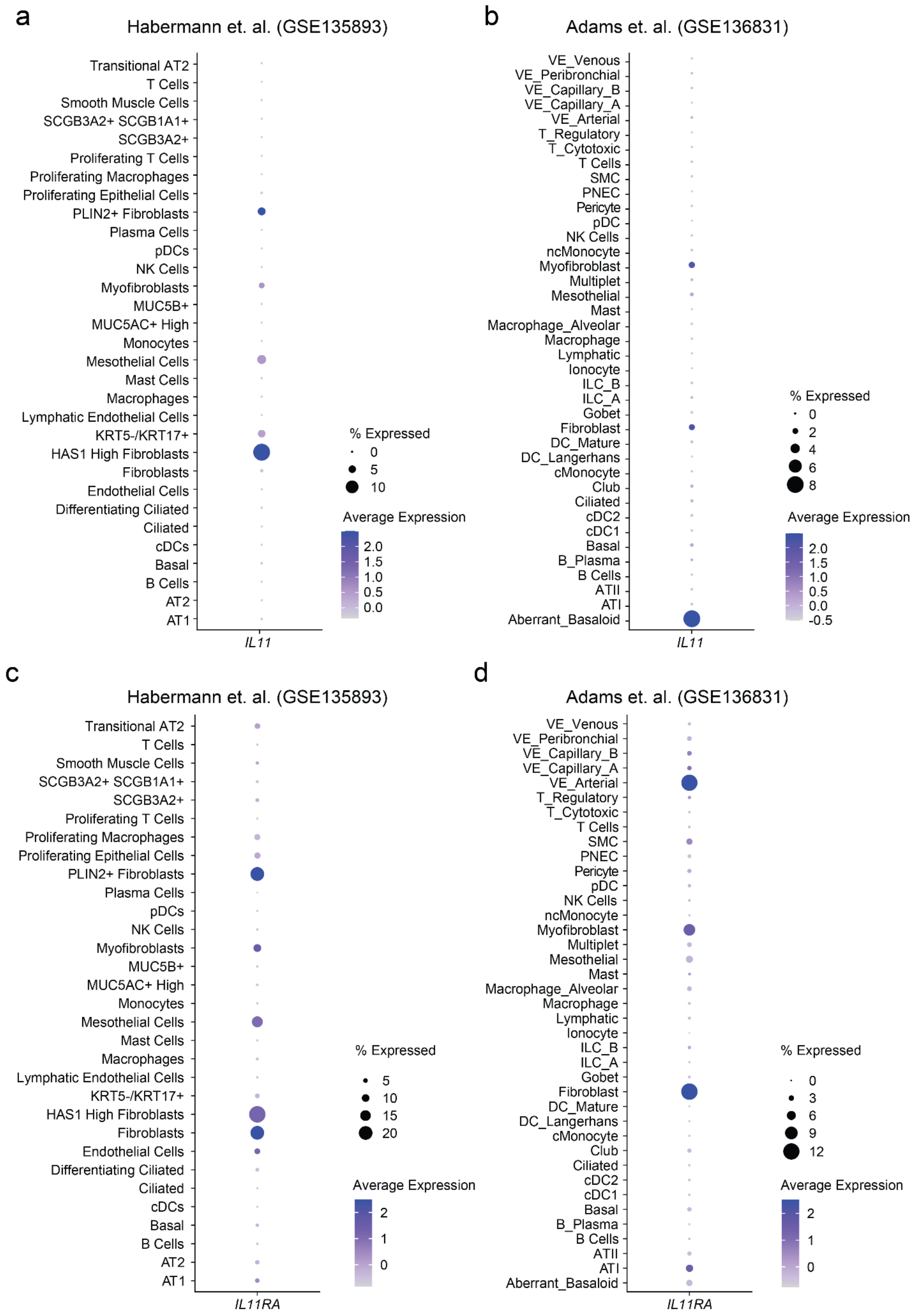

Supplementary Figure 3. The expression profile of *IL11* and *IL11RA* across various cell types in the human lung. Dot-plots showing the expression of *IL11* or *IL11RA* in individual cell types in scRNA-seq data from control and PF samples in the Habermann et. al. (GSE135893) and Adams et. al. (GSE136831) datasets.

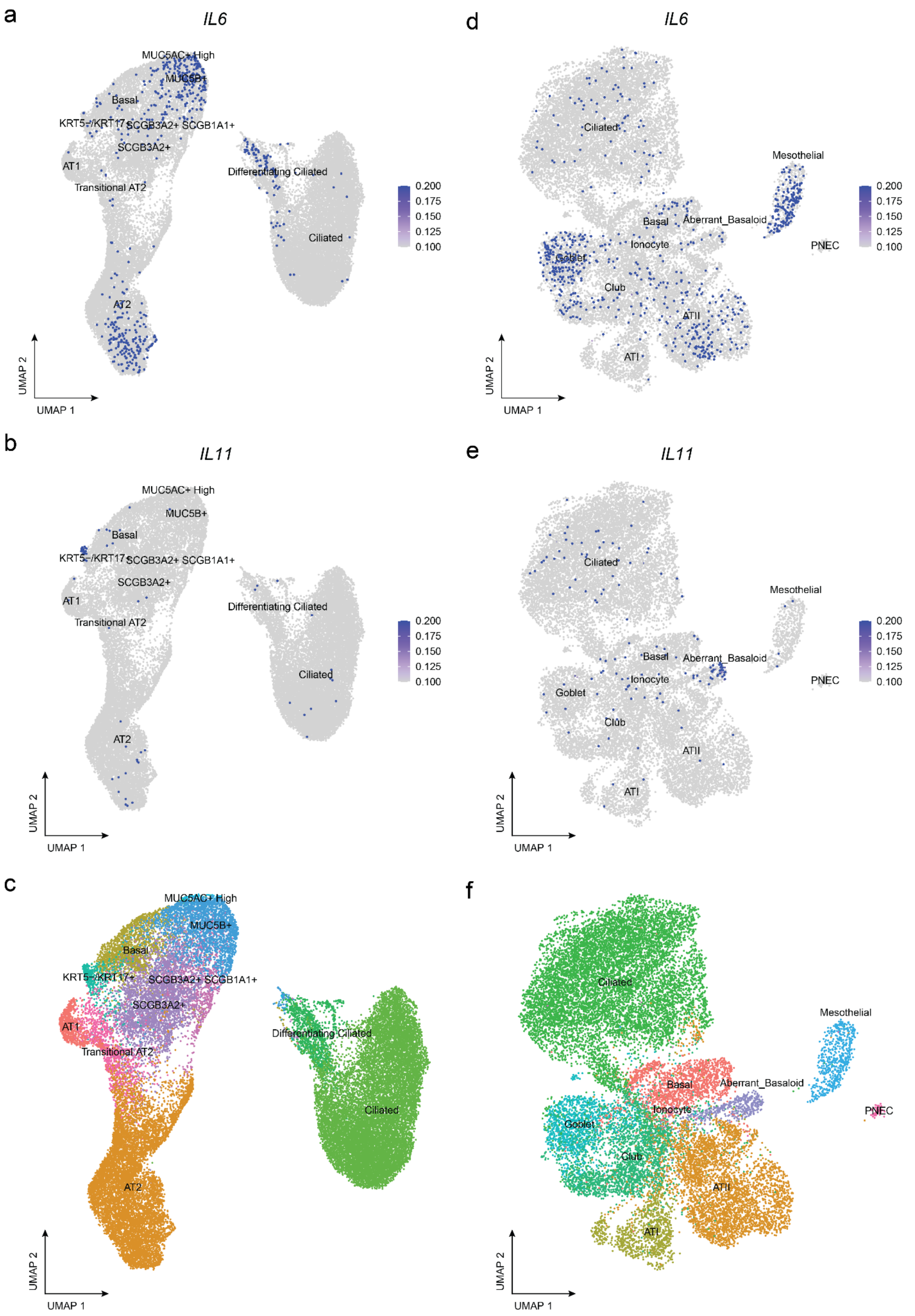

Supplementary Figure 4. *IL6* and *IL11* expression in various epithelial cell types in the human lung. UMAP visualization of *IL6* or *IL11* expressing single cells in scRNA-seq data from control and PF samples in the (**a-c**) Habermann et. al. (GSE135893) and (**d-f**) Adams et. al. (GSE136831) dataset. *IL6 or IL11* expressing cells are colored in dark blue in **a**, **b**, **d**, **e** and colored dots indicate different cell clusters in **c** and **f**.

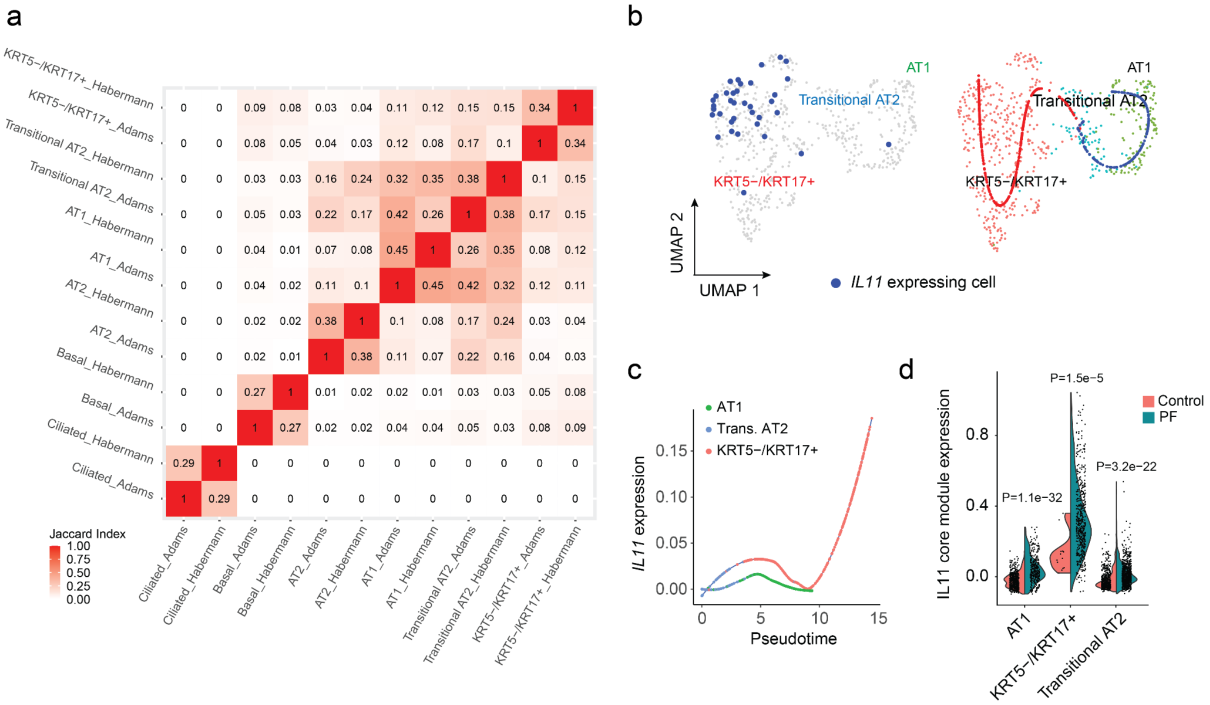

Supplementary Figure 5. ***IL11*** **is expressed by aberrant basaloid cells in human pulmonary fibrosis.** (**a**) Heatmap showing the transcriptional similarities between selected epithelial cell types from Habermann et. al., (GSE135893) and Adams et. al. (GSE136831) datasets as assessed by the Jaccard index. (**b**) UMAP visualization of *IL11* expressing single cells in the Adams et. al. dataset. *IL11* expressing cells are colored in dark blue (left panel) and colored dots indicate cell type clustering (right panel). Cell labels were assigned using the classification from Habermann et. al. by Seurat's FindTransfer Algorithm (see Methods). Blue line indicates differentiation trajectory from transitional AT2 to AT1 cells; red line indicates differentiation trajectory from transitional AT2 to KRT5$^-$/KRT17$^+$. Data are composed of PF samples in the Adams et. al. dataset. (**c**) Expression of *IL11* in the pseudotime trajectory from transitional AT2 to KRT5$^-$/KRT17$^+$ versus from transitional AT2 to AT1 cells. Data are composed from PF samples in the Adams et. al. dataset. (**d**) Expression of *IL11* co-expression module in transitional AT2, KRT5$^-$/KRT17$^+$ and AT1 cells from control versus PF in combined Habermann et. al. and Adams et. al. datasets.

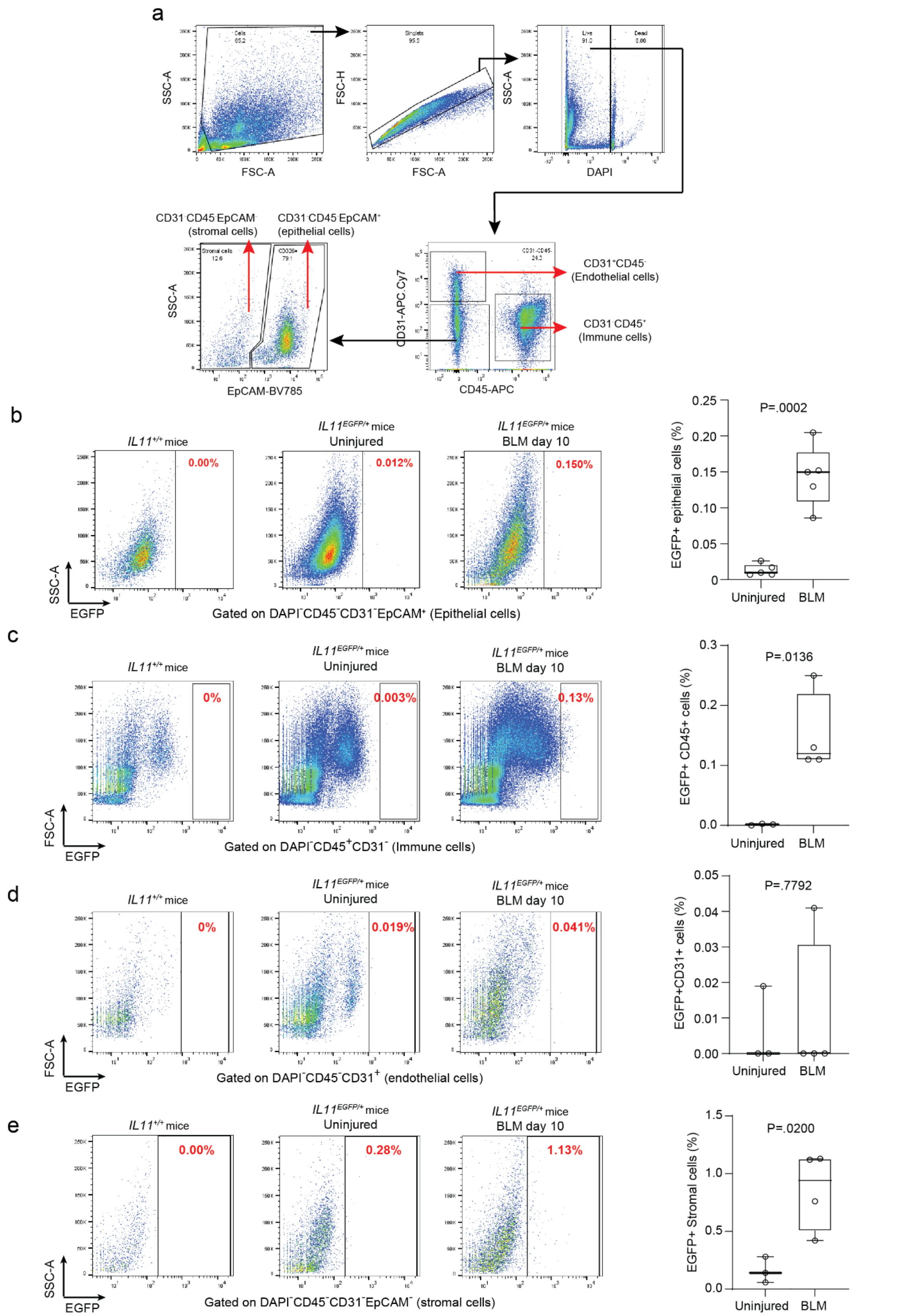

Supplementary Figure 6. Flow cytometry gating of endogenous EGFP expressing cells from *IL11*^{EGFP} reporter mice lungs after bleomycin injury. (**a**) Representative gating for flow cytometry analysis of lung stromal and epithelial cells isolated from *IL11*^{EGFP} reporter mice based on antibody staining for CD31, CD45 or CD326 (EpCAM). The predetermined baseline thresholds for endogenous IL11^{EGFP+} expression in each respective cell type were based on the EGFP signal from *IL11*$^{+/+}$ lung cells. The percentages of (**b**) EGFP^{+}

epithelial cells ($CD31^{-}CD45^{-}EpCAM^{+}$) (**c**) $EGFP^{+}$ immune cells ($CD31^{-}CD45^{+}$), (**d**) $EGFP^{+}$ endothelial cells ($CD31^{+}CD45^{-}$) or (**e**) $EGFP^{+}$ stromal cells ($CD31^{-}CD45^{-}EpCAM^{-}$) in uninjured and bleomycin (BLM)-treated $IL11^{EGFP}$ reporter mice. *P* value determined by two-tailed Student's *t*-test, n = 3 controls and 4 BLM-treated mice / group. Data are represented as median ±IQR and whiskers extend from minimum to maximum values.

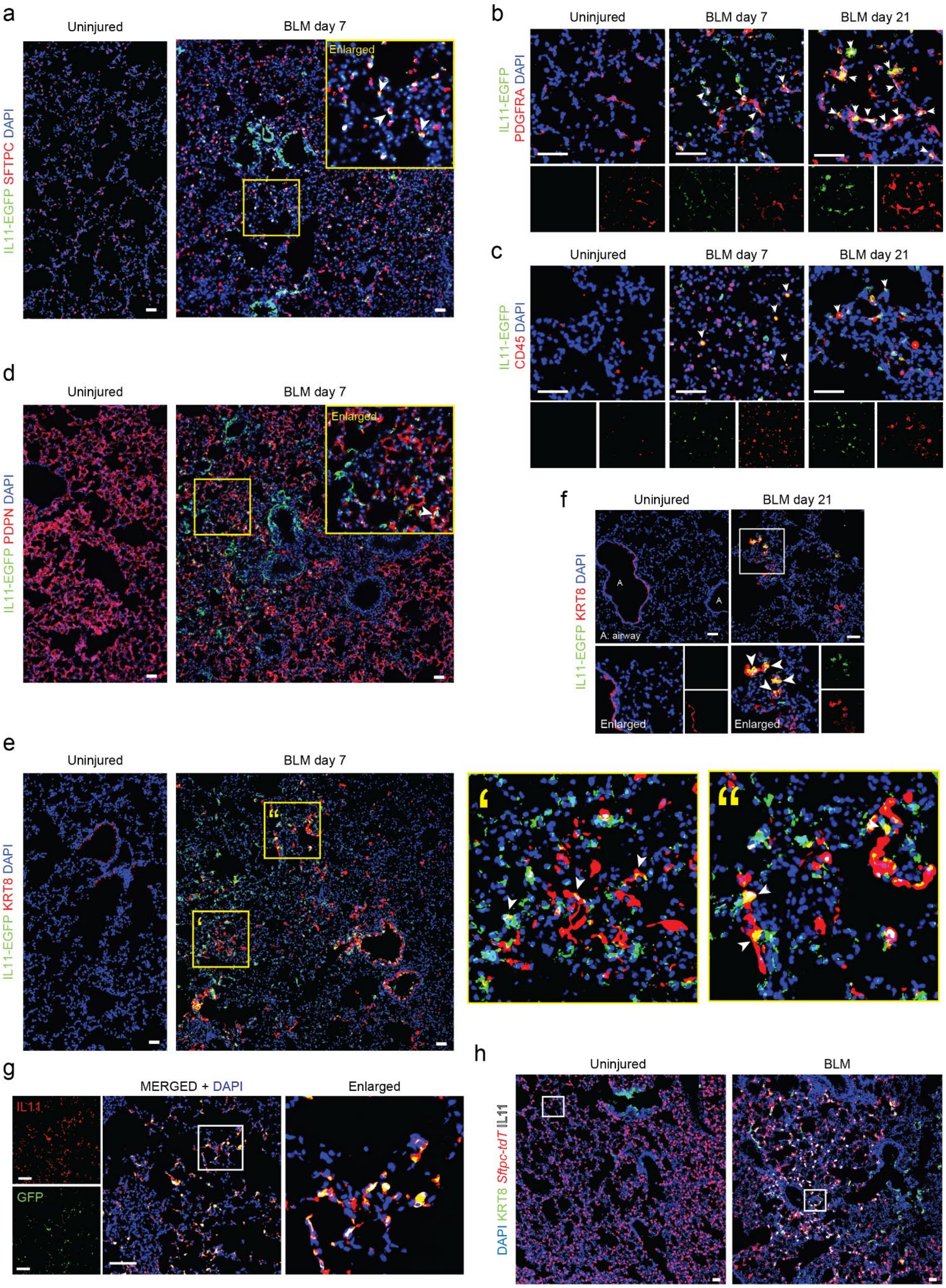

Supplementary Figure 7. IL11 is upregulated in alveolar epithelial cells, fibroblasts and CD45^{+} cells after bleomycin-induced lung injury in mice. Immunostaining for GFP and (**a**) SFTPC or (**b**) PDGFRA or (**c**) CD45 or (**d**) PDPN or (**e-f**) KRT8 in injured alveolar regions of *IL11^{EGFP}* reporter mice post-BLM injury Scale bars: 50 µm. White arrowheads indicate marker positive *IL11^{EGFP+}* cells. (**g**) Immunostaining of lungs from *IL11^{EGFP}* reporter mice post-BLM injury with anti-GFP and anti-IL11 antibodies, showing considerable overlap

of GFP and IL11 signals. Scale bars: 100 µm. (**h**) Immunostaining of KRT8 and IL11 in lungs from AT2 lineage reporter (*Sftpc-tdT*) mice post-BLM injury. Scale bars: 50 µm. White boxes in **h** indicate enlarged regions shown in Fig. 2k. Data are representative of at least 3 independent experiments.

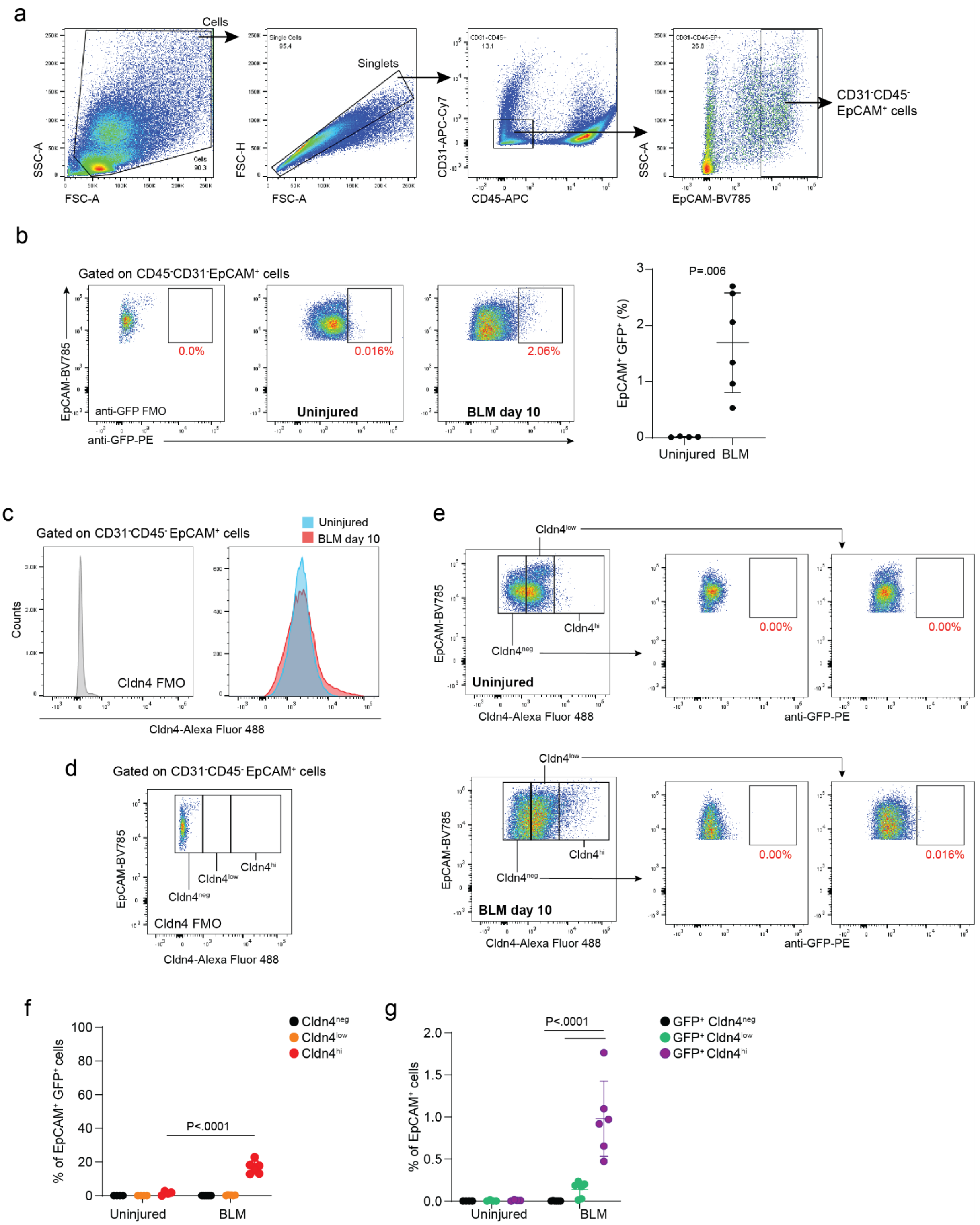

Supplementary Figure 8. Flow cytometry analysis of GFP and Cldn4 expression in lung epithelial cells from *IL11^{EGFP}* reporter mice after bleomycin-induced lung injury. (**a**) Flow cytometry gating strategy for lung epithelial cells (CD31$^-$ CD45$^-$ EpCAM$^+$). (**b**) Flow cytometry analysis and quantification of IL11-expressing lung epithelial cells from *IL11^{EGFP}* reporter mice post-BLM injury by gating for GFP$^+$ and EpCAM$^+$ cells. Fluorescence minus one (FMO) control for gating of GFP$^+$ signal is shown in the left panel. (**c**) Histogram plot of cell counts and Cldn4-Alexa Fluor 488 signal in lung epithelial cells from uninjured and BLM-injured *IL11^{EGFP}* reporter mice. (**d**) FMO control for the gating of Cldn4$^+$ signal and the general gating strategy for delineating Cldn4^{hi}, Cldn4^{low} and Cldn4$^{neg.}$ cell populations. (**e**) Flow cytometry analysis of GFP$^+$ cells in Cldn4^{low} and Cldn4$^{neg.}$ cells from uninjured and BLM-injured *IL11^{EGFP}* reporter mice. (**f**) The percentage of

GFP-expressing cells across individual Cldn4^{hi}, Cldn4^{low} or Cldn4$^{neg.}$ cell subsets. (**g**) The percentage of EpCAM^{+} cells constituted by the various GFP^{+} Cldn4 subsets. Data are represented as mean ± s.d. *P* values were determined by two-tailed Student's *t*-test in **b**, by two-way ANOVA in **f** or by One-way ANOVA in **g**, n = 4 control and 6 BLM-treated mice / group.

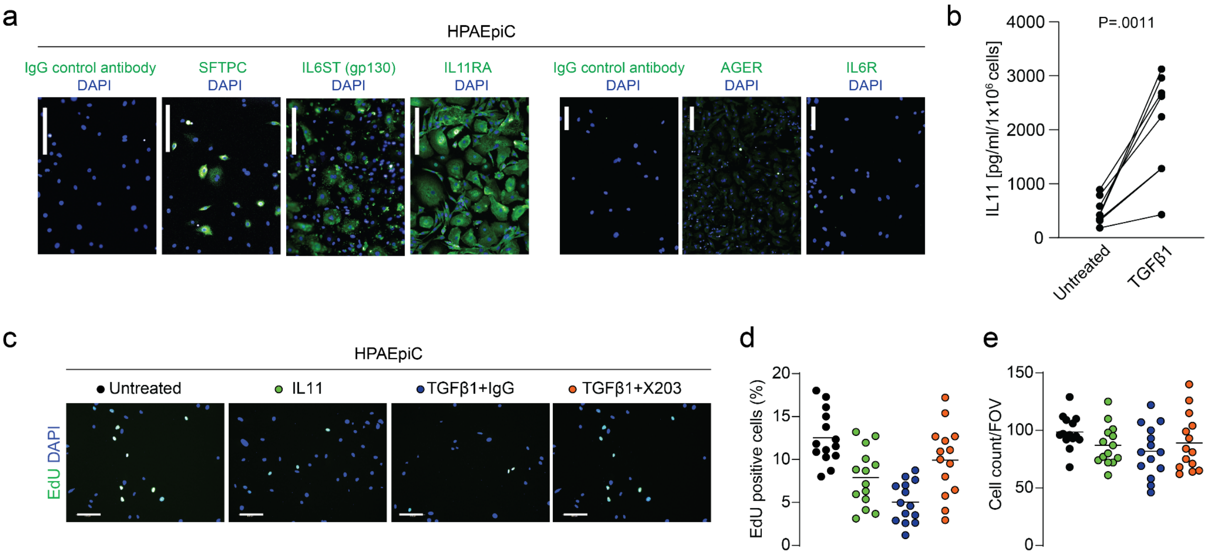

Supplementary Figure 9. TGFβ induces IL11 secretion by alveolar epithelial cells and IL11 reduces alveolar epithelial cell proliferation *in vitro*. (**a**) Representative images of immunostaining for SFTPC, AGER, IL6ST (gp130), IL11RA and IL6R in primary human alveolar epithelial cells (HPAEpiC). Scale bars: 200 µm. (**b**) ELISA-based quantification of IL11 in culture supernatant of TGFβ1-treated HPAEpiC (5 ng/ml, 24 hours, n = 6). (**c-d**) Representative images and quantification of EdU incorporation and (**e**) mean cell counts per field of view (FOV) in HPAEpiCs treated with IL11 (5 ng/ml), TGFβ1 (5 ng/ml) in the presence of anti-IL11 (X203) or IgG control antibodies (2 µg/ml). One representative dataset from two independent biological experiments are shown (mean fluorescence intensity/area for 14 non-overlapping fields per condition are shown). Scale bars: 100 µm. *P* values were determined by two-tailed paired Student's *t*-test in **b**.

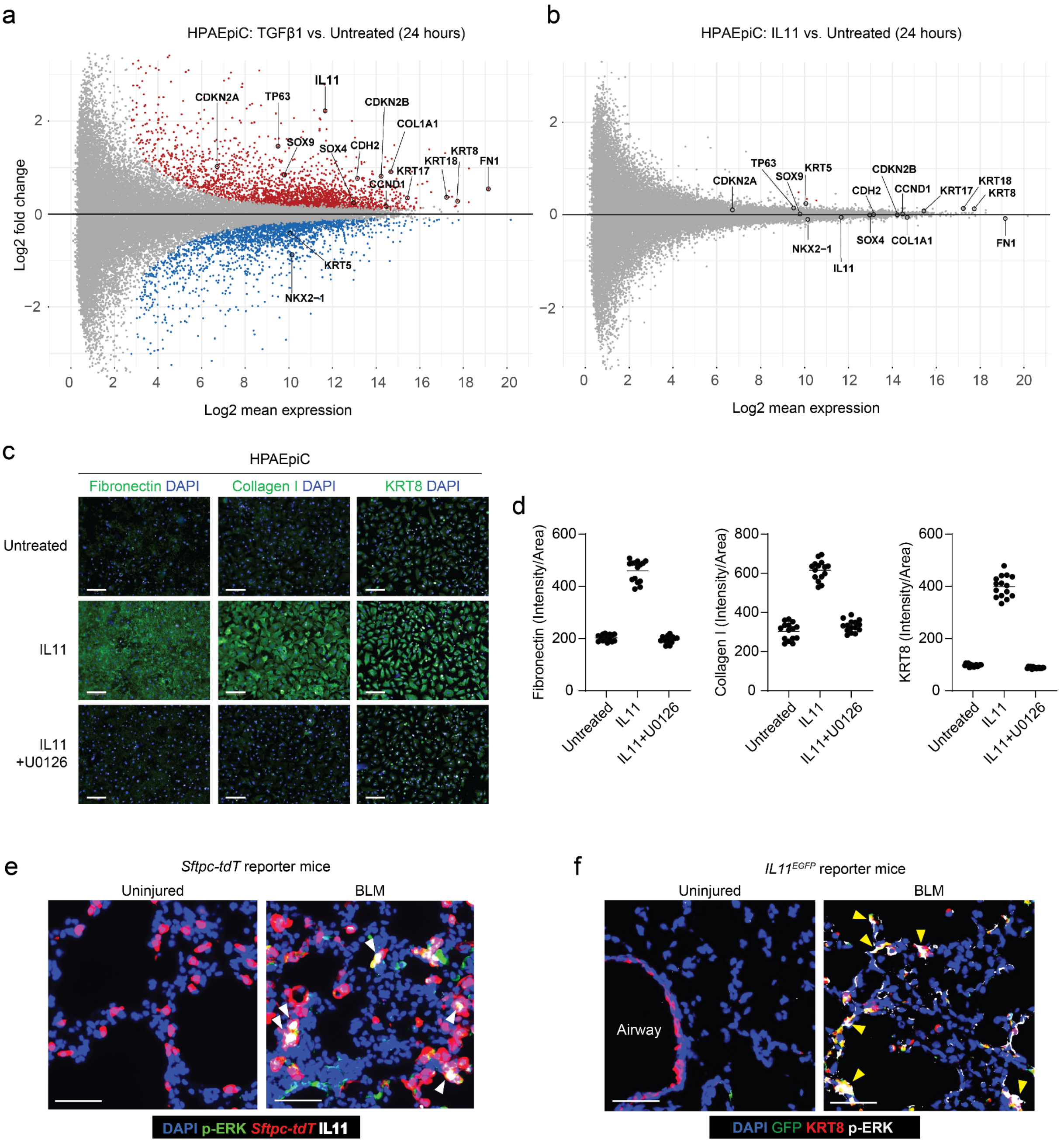

Supplementary Figure 10. IL11-dependent ERK signaling promotes ECM-related protein expression by alveolar epithelial cells *in vitro* and p-ERK is induced in IL11-expressing transitional alveolar epithelial cells after bleomycin-induced lung injury. (**a-b**) RNA-seq analysis of TGFβ1- or IL11-treated (5 ng/ml, 24 hours) HPAEpiC. Red and blue dots indicate significantly differentially upregulated or downregulated genes respectively (FDR < 0.05). (**c-d**) Representative images and quantification of immunostaining of Fibronectin, Collagen I and KRT8 in HPAEpiC treated with IL11 (5 ng/ml) in the presence/absence of MEK inhibitor U0126 (10 µM) for 24 hours. Scale bars: 200 µm. One representative dataset from three independent biological experiments are shown (mean fluorescence intensity/area for 14 non-overlapping fields per condition are shown). (**e**) Representative images of immunostaining for IL11 and p-ERK in the lungs of *Sftpc-tdT* reporter mice post-BLM injury. Scale bars: 50 µm. White arrowheads indicate p-ERK$^+$ IL11$^+$ *tdT*$^+$ cells. (**f**) Representative images of immunostaining for GFP, KRT8 and p-ERK in the lungs of *IL11*^{EGFP} reporter mice post-BLM injury. Scale bars: 50 µm. Yellow arrowheads indicate p-ERK$^+$ GFP$^+$ KRT8$^+$ cells. Data in **e** and **f** are representative of three independent experiments.

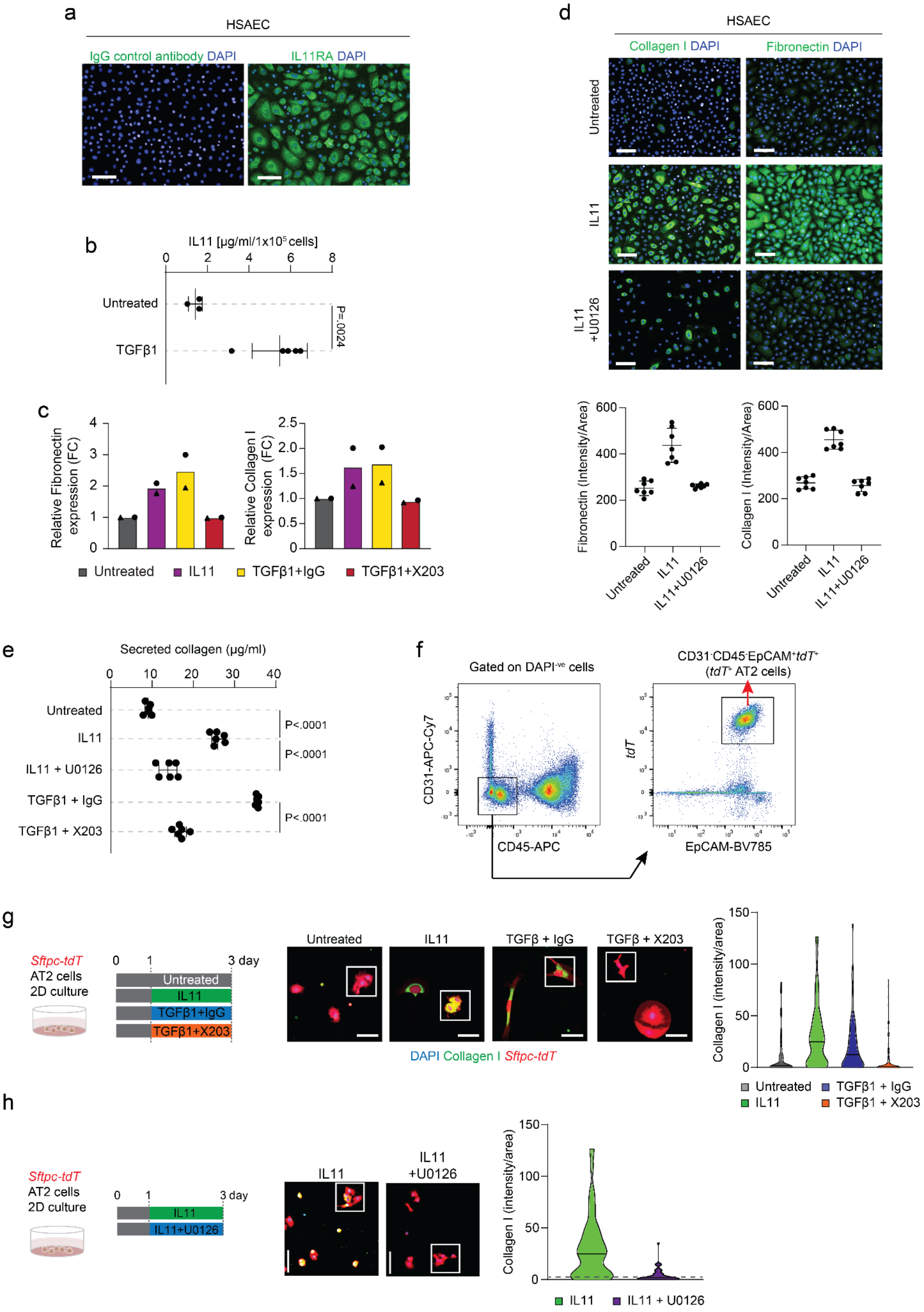

Supplementary Figure 11. IL11-dependent ERK signaling promotes ECM-related protein expression by human small airway epithelial cells and mouse AT2 cells *in vitro*. (**a**) Representative images of immunostaining for IL11RA in primary human small airway epithelial cells (HSAEC). (**b**) ELISA-based

quantification of IL11 in culture supernatant of TGFβ1-treated HSAEC (5 ng/ml, 24 hours). Data are representative of three independent experiments and represented as mean ± s.d. *P* values were determined by two-tailed student's *t* test. (**c**) Immunofluorescence quantification of Fibronectin and Collagen I expression of HSAEC treated with IL11 (5 ng/ml) or TGFβ1 (5 ng/ml) in the presence of anti-IL11 (X203) or IgG control antibodies (2 μg/ml) for 24 hours; n = 2. FC: fold change. (**d**) Representative images and immunofluorescence quantification for Collagen I or fibronectin in HSAEC treated with IL11 (5 ng/ml) in the presence/absence of MEK inhibitor U0126 (10 μM) for 24 hours. Scale bars: 100 μm. Data are representative of two independent experiments (mean fluorescence intensity/area for 7 non-overlapping fields per condition are shown). (**e**) Secreted collagen concentration from HSAEC treated with various stimuli for 24 hours. Data are represented as mean ± s.d. *P* values were determined by one-way ANOVA (Tukey's test). (**f**) Gating for FACs sorting of AT2 cells ($CD45^-$ $CD31^-$ $EpCAM^+$ tdT^+) from *Sftpc-tdT* mice. (**g-h**) Representative images of immunostaining and quantification of Collagen I expression in tdT^+ AT2 cells from *Sftpc-tdT* reporter mice treated with (**g**) IL11 (5 ng/ml), TGFβ1 (5 ng/ml) in the presence of anti-IL11 (X203) or IgG control antibodies (2 μg/ml) or (**h**) IL11 (5 ng/ml) in the presence/absence of MEK inhibitor U0126 (10 μM) for 48 hours. Scale bars: 50 μm. White boxes in panels **g** and **h** indicate enlarged images shown in **Fig. 3j** and **3l** respectively. Data in **g** and **h** are representative and are pooled from two independent experiments (n ≥ 50 cells / group).

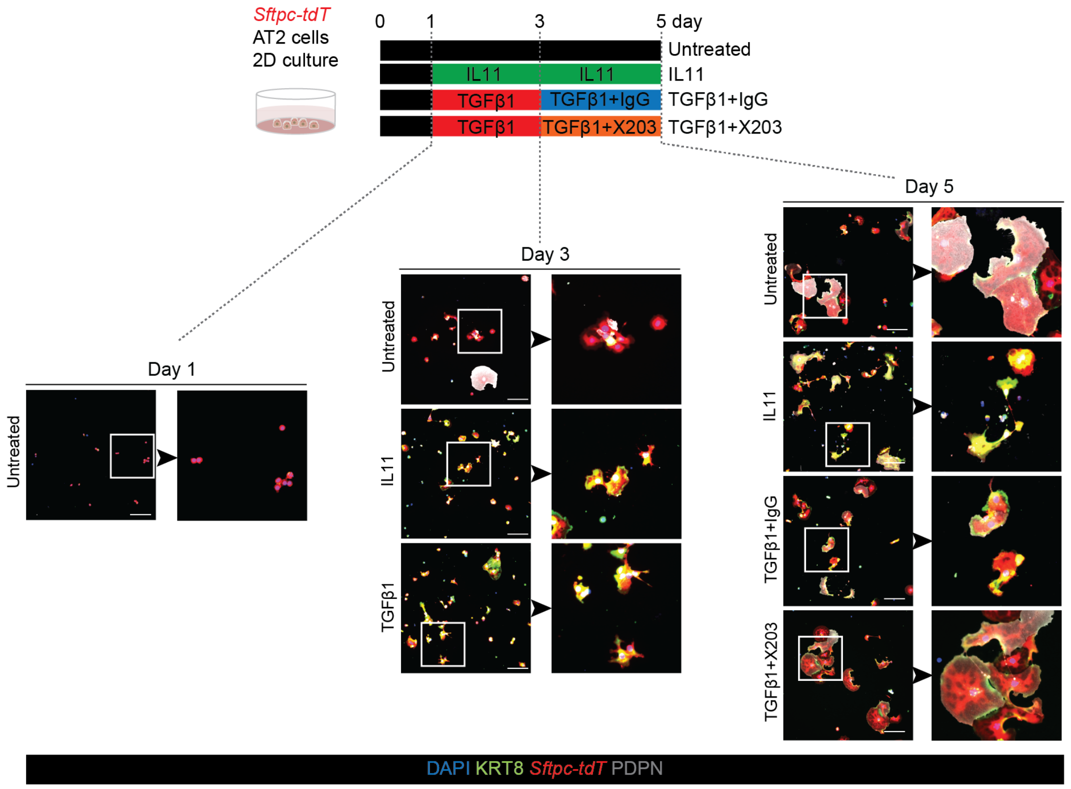

Supplementary Figure 12. IL11 impairs AT2-to-AT1 differentiation of mouse AT2 cells in vitro. Time series (day 1 to 5) of *Sftpc-tdT*$^+$ AT2 cells treated with IL11 (5 ng/ml), TGFβ1 (5 ng/ml), X203 or IgG control antibodies (2 μg/ml). tdT^+ cells (red) were immunostained for KRT8 (green), PDPN (white) and counterstained with DAPI (blue). Scale bars: 50 μm. Data are representative of two independent experiments.

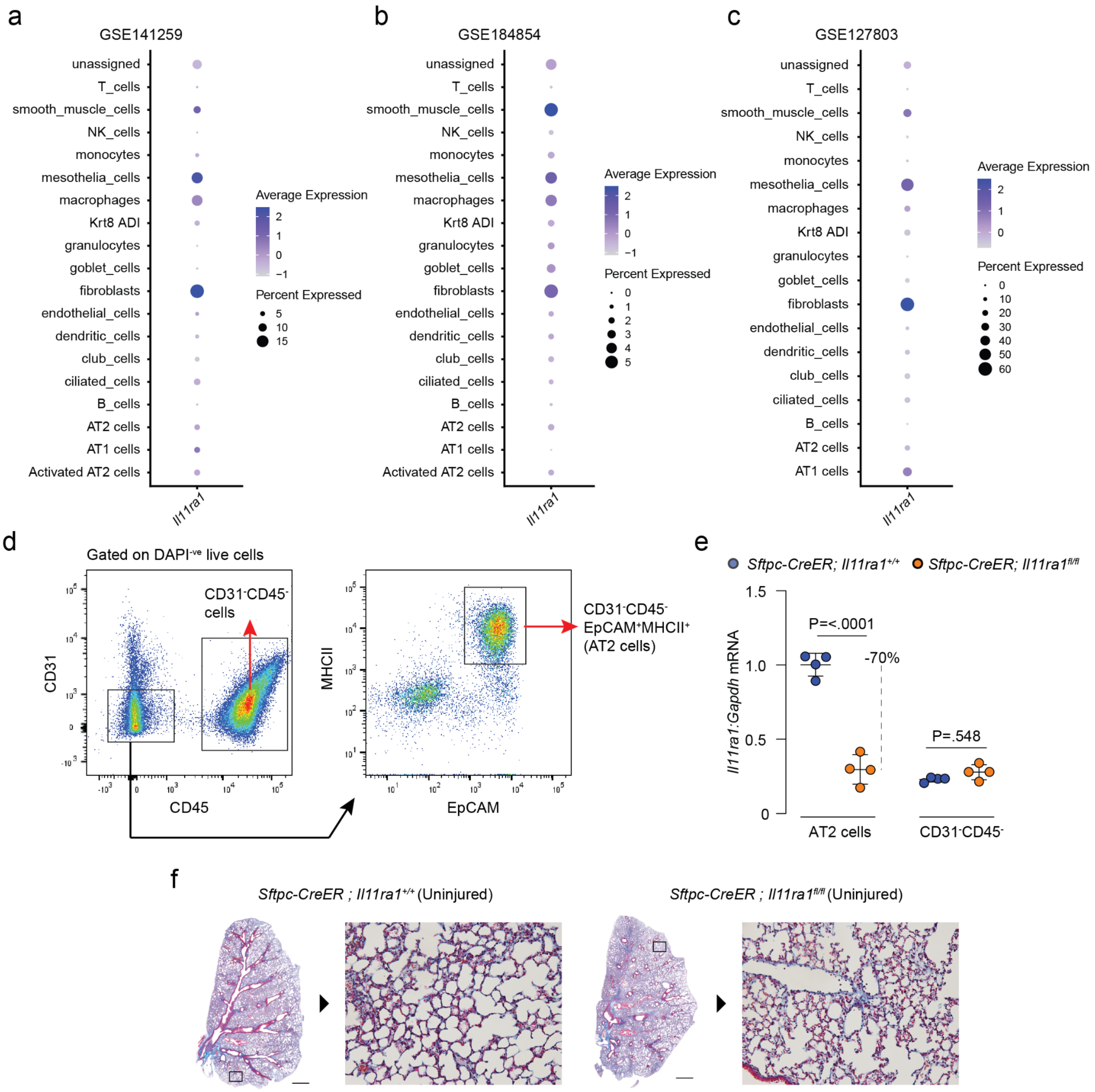

Supplementary Figure 13. Characterization of AT2 cell-specific *Il11ra1*-deleted mice after bleomycin-induced lung injury. (**a-c**) Dot-plots showing the expression of *Il11ra1* across various mouse lung cell types in scRNA-seq datasets from (**a**) Strunz et. al. (GSE141259), (**b**) Ogawa et al. (GSE184854) and (**c**) Joshi et al. (GSE127803). (**d**) Gating for FACs sorting of CD45^{+} CD31^{-} or CD45^{-} CD31^{-} EpCAM^{+} MHCII^{+} lung cells from *Sftpc-CreER; Il11ra1*$^{fl/fl}$ mice. (**e**) qPCR analysis of *Il11ra1* expression in AT2 cells (CD45^{-} CD31^{-} EpCAM^{+} MHCII^{+}) and CD45^{+} immune cells isolated from the lungs of *Sftpc-CreER; Il11ra1*$^{fl/fl}$ or *Sftpc-CreER; Il11ra1*$^{+/+}$ mice. n = 4 mice / group. (**f**) Images of Masson's trichrome staining of lungs in uninjured *Sftpc-CreER; Il11ra1*$^{fl/fl}$ *or Il11ra1*$^{+/+}$ mice. Scale bars: 1000 µm.

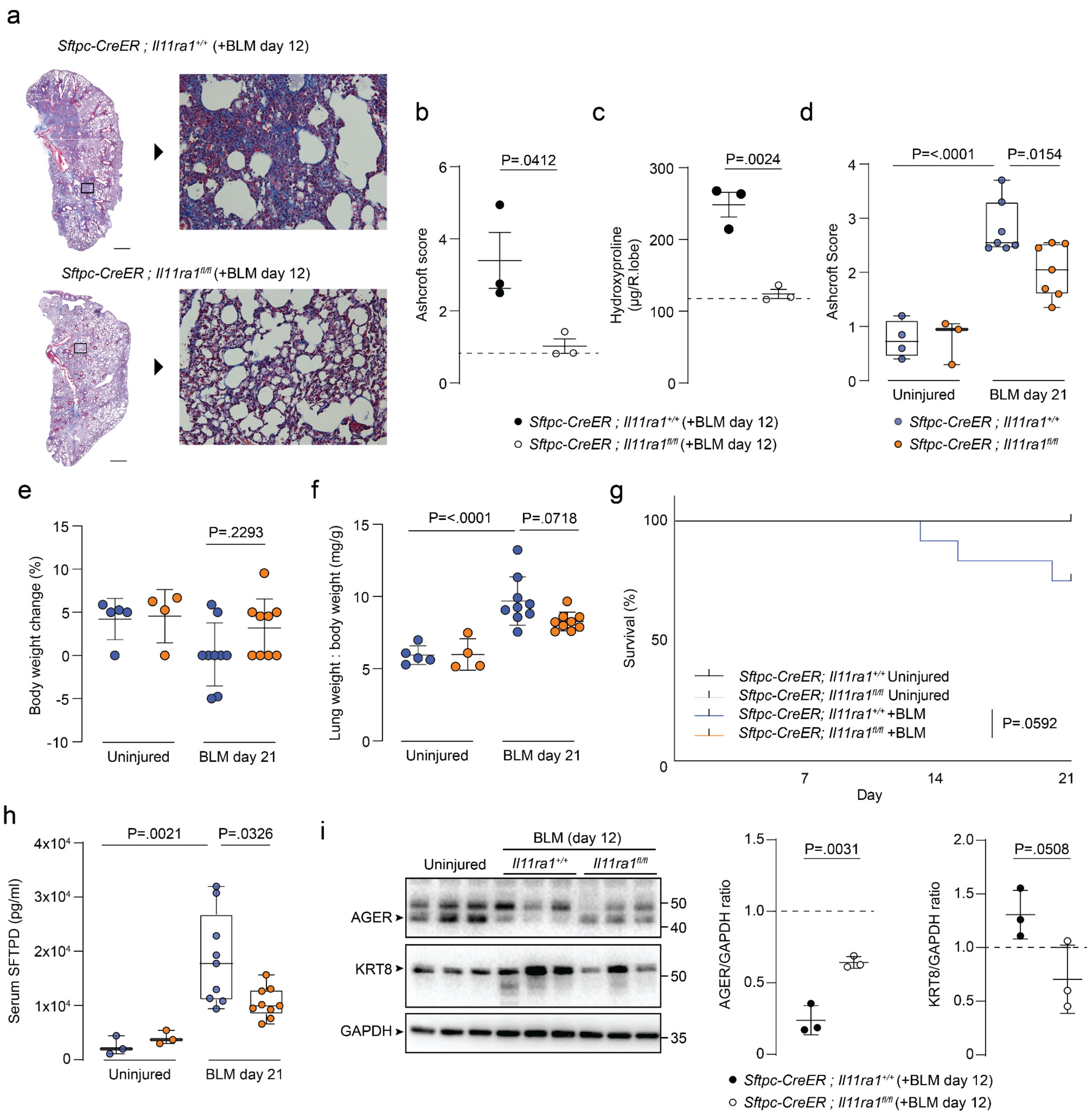

Supplementary Figure 14. AT2 cell-specific *Il11ra1*-deleted mice are protected against bleomycin-induced lung fibrosis. (**a**) Images of Masson's trichrome staining of lungs 12 days post-BLM injury, (**b**) lung histopathological fibrosis scores and (**c**) lung hydroxyproline content of right caudal lobes from *Sftpc-CreER; Il11ra1$^{fl/fl}$ or Il11ra1$^{+/+}$* mice. n = 3 mice / group. Scale bars: 1000 µm. Dashed lines indicate mean values of uninjured controls. (**d**) Lung histopathological fibrosis scores (**e**) Percentage body weight change post-BLM injury (day 21 versus day 0), (**f**) Lung weight to body weight indices and (**g**) survival analysis and (**h**) ELISA-based quantification of serum SFTPD from *Sftpc-CreER; Il11ra1$^{fl/fl}$ or Il11ra1$^{+/+}$* mice 21 days post-BLM treatment. n = 3-4 controls and 7-9 BLM-treated mice / genotype. (**i**) Western blot and densitometry analysis of AGER and KRT8 expression in lung homogenates from *Sftpc-CreER; Il11ra1$^{fl/fl}$ or Il11ra1$^{+/+}$* mice 12 days post-BLM injury. n = 3 mice / group. KRT8 (~55 kDa band) and AGER protein (~43 kDa band) were quantified by densitometry. Dashed lines indicate mean values of uninjured controls. GAPDH control was performed on different membranes. Uncropped blots are shown in Supplementary Figure 24. Data are represented as mean ± s.d. *P* values were determined by two tailed Student's *t*-test panel **b, c** and **i** and by one-way ANOVA (Tukey's test) in panels **d, f** and **h** and by Log-rank (Mantel-Cox) test in panel **g**.

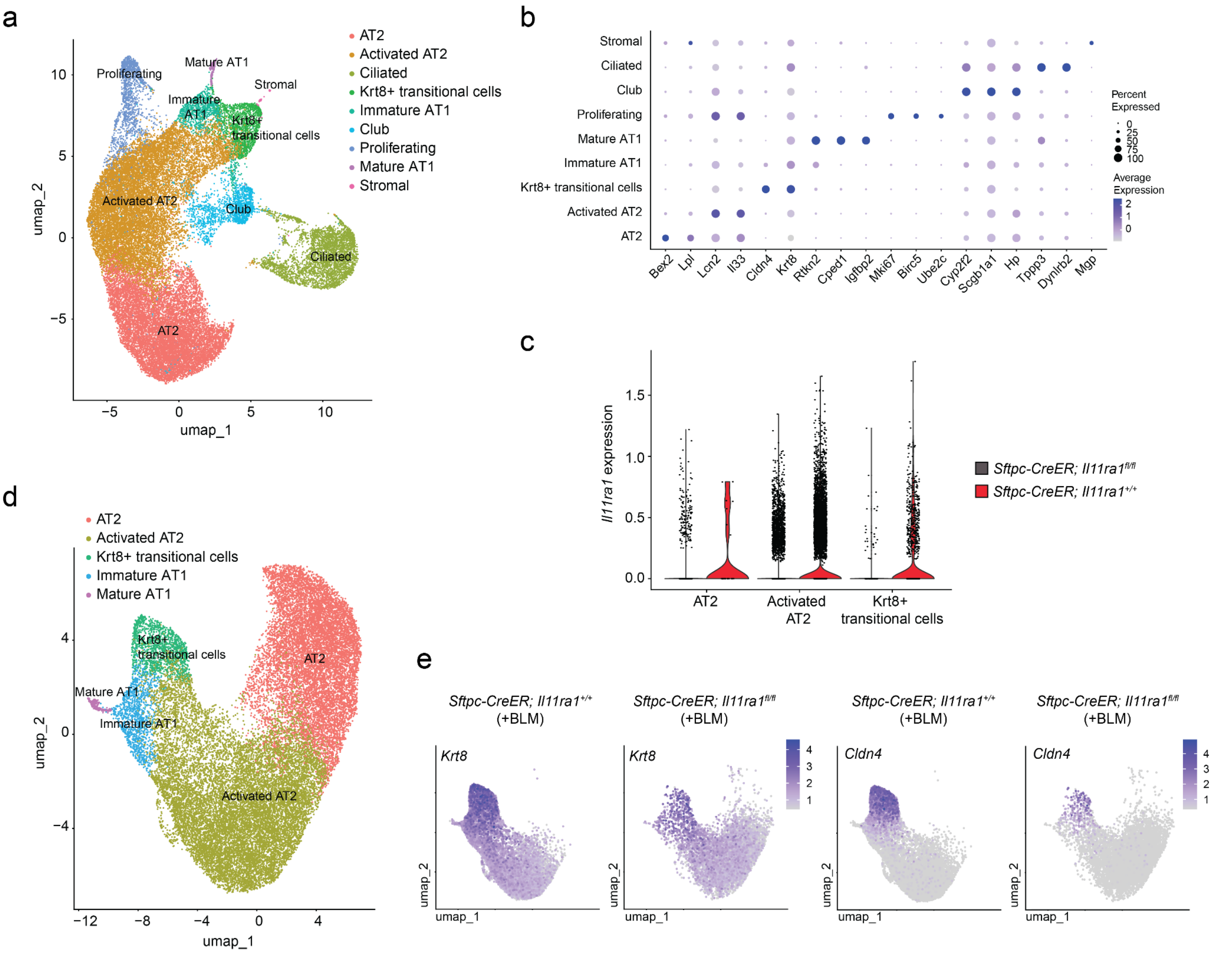

Supplementary Figure 15. scRNA-seq analysis of epithelial cells from AT2 cell-specific *Il11ra1*-deleted mice after bleomycin-induced lung injury. (**a**) UMAP visualization of lung epithelial cells colored by cell identity (K = 36,443 cells). (**b**) Dot plot showing the expression of various cell identity markers. (**c**) violin plot showing the relative expression of *Il11ra1* in various AT2-lineage cells from *Sftpc-CreER; Il11ra1*$^{fl/fl}$ or *Il11ra1*$^{+/+}$ mice. (**d**) UMAP visualization of AT2-lineage and AT1 cells and (**e**) UMAP visualization of *Krt8* or *Cldn4* expressing single cells (purple dots).

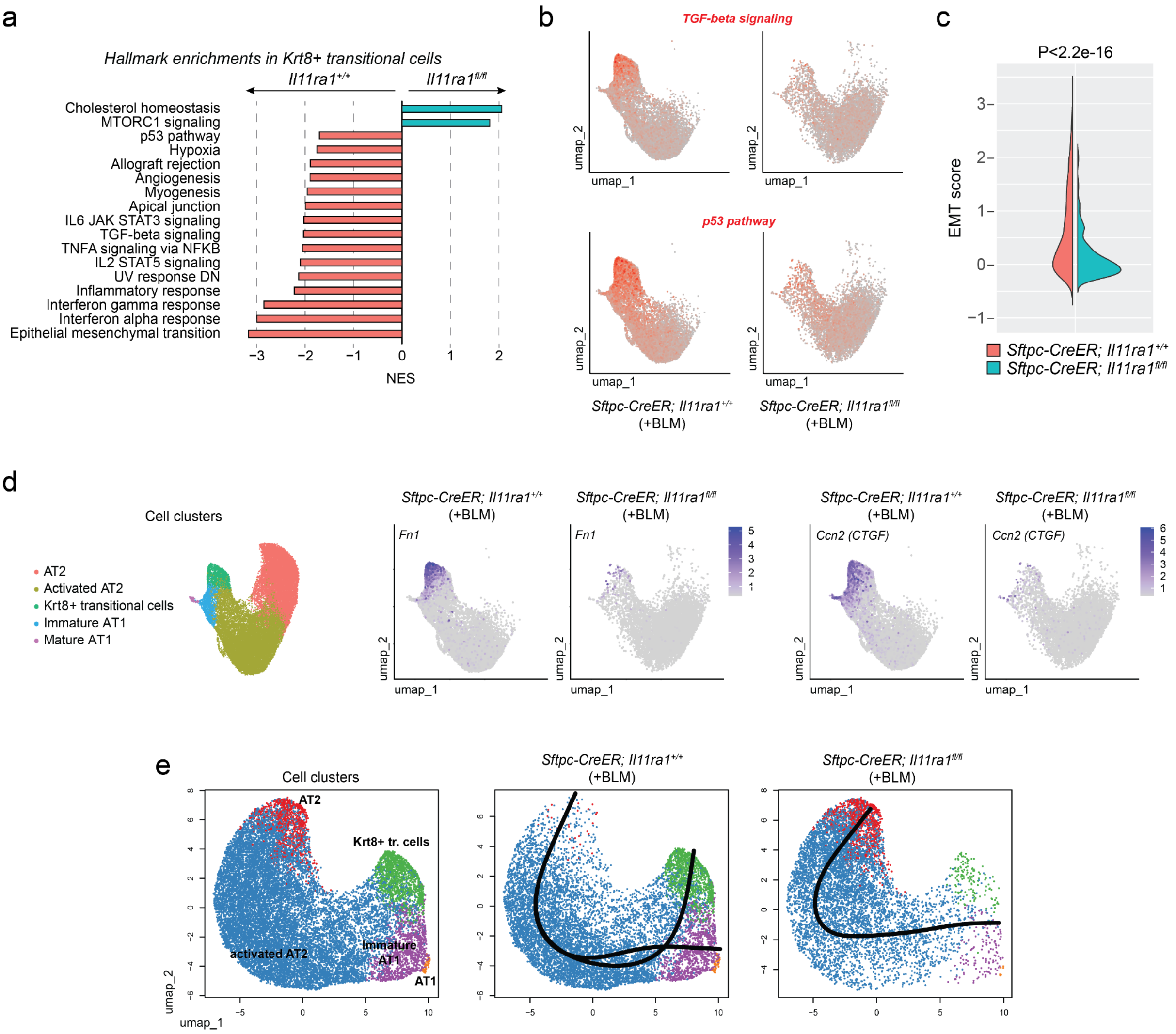

Supplementary Figure 16. AT2 cell-specific IL11-signaling promotes pathological transcriptional programs in transitional epithelial cells after bleomycin-induced lung injury. (**a**) Normalized enrichment scores (NES) of pathways significantly enriched in Krt8+ transitional cells and (**b**) single cells colored by gene expression signature scores for the indicated pathways from the MSigDB Hallmark gene sets of AT2-lineage cells from *Sftpc-CreER; Il11ra1*$^{fl/fl}$ or *Il11ra1*$^{+/+}$ mice post-BLM challenge. (**c**) Violin plot visualization of EMT score based on leading edge analysis of EMT Hallmark (GSEA) genes in Krt8+ transitional cells and (**d**) UMAP visualization of *Fn1* or *Ccn2* (encodes for CTGF) expressing single cells (purple dots) from AT2 lineage cells from *Sftpc-CreER; Il11ra1*$^{fl/fl}$ or *Il11ra1*$^{+/+}$ mice post-BLM challenge. (**e**) UMAP embedding of Slingshot-based trajectory of AT2, activated AT2, Krt8+ transitional cells and AT1 cells from *Sftpc-CreER; Il11ra1*$^{fl/fl}$ or *Il11ra1*$^{+/+}$ mice lungs post-BLM challenge. *P* value in **c** was determined by Wilcoxon signed-rank test.

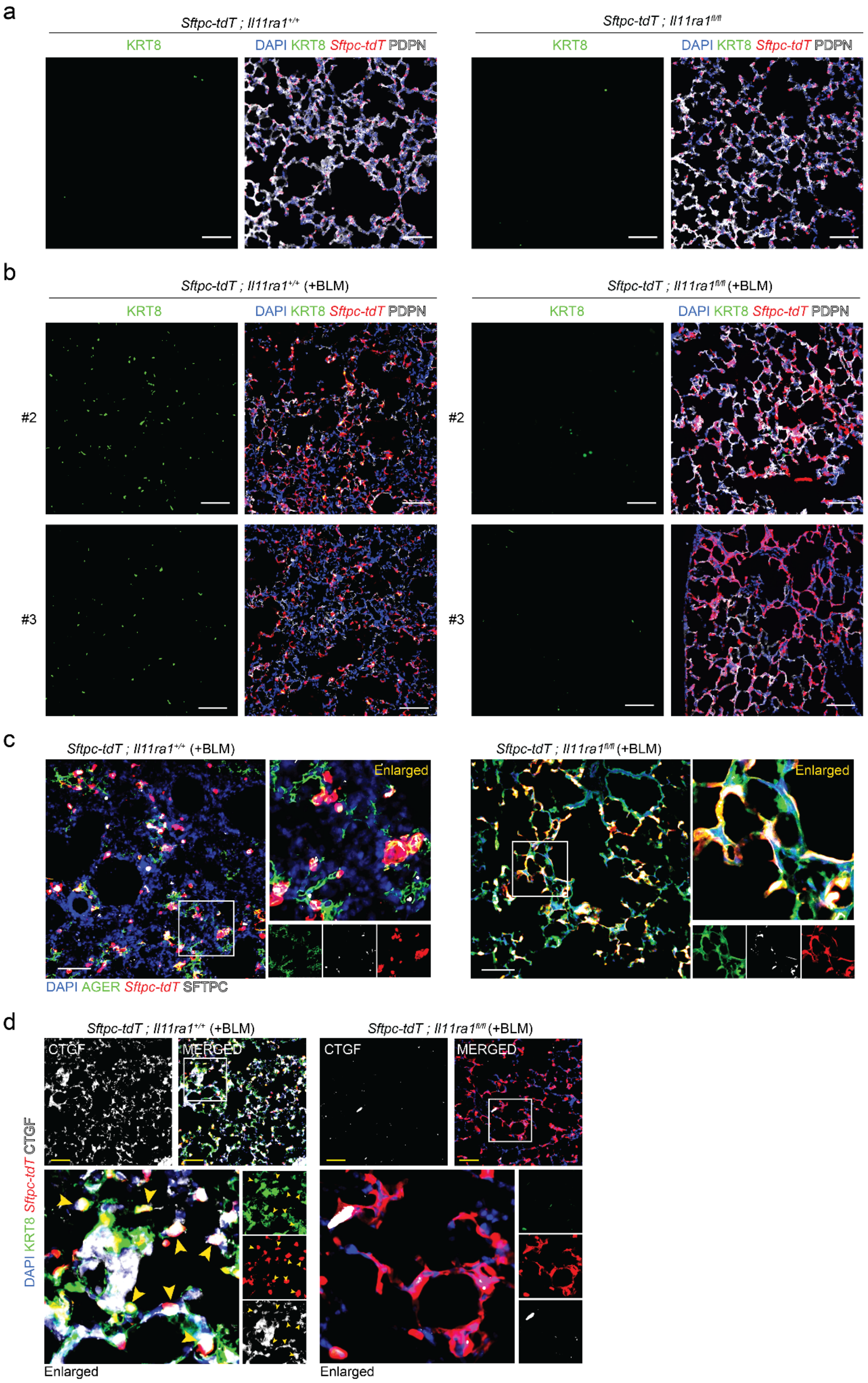

Supplementary Figure 17. AT2 cell-specific *Il11ra1*-deleted mice exhibit reduced accumulation of profibrotic transitional epithelial cells and enhanced alveolar epithelial repair after bleomycin-induced lung injury. Images of immunostaining for KRT8 and PDPN in lungs of (**a**) uninjured or (**b**) BLM injured (day 12) *Sftpc-tdT; Il11ra1*$^{fl/fl}$ *or Il11ra1*$^{+/+}$ mice. Various replicates used for quantification data in Fig. 4k and i are shown in **b**. Images of immunostaining for (**c**) AGER and SFTPC or (**d**) KRT8 and CTGF in lungs of *Sftpc-tdT; Il11ra1*$^{fl/fl}$ *or Il11ra1*$^{+/+}$ mice 12 days post-BLM injury. Scale bars: 100 µm. Yellow arrowheads in **d** indicate KRT8^{+} *tdT*$^{+}$ CTGF^{+} cells. Data are representative of at least three independent experiments.

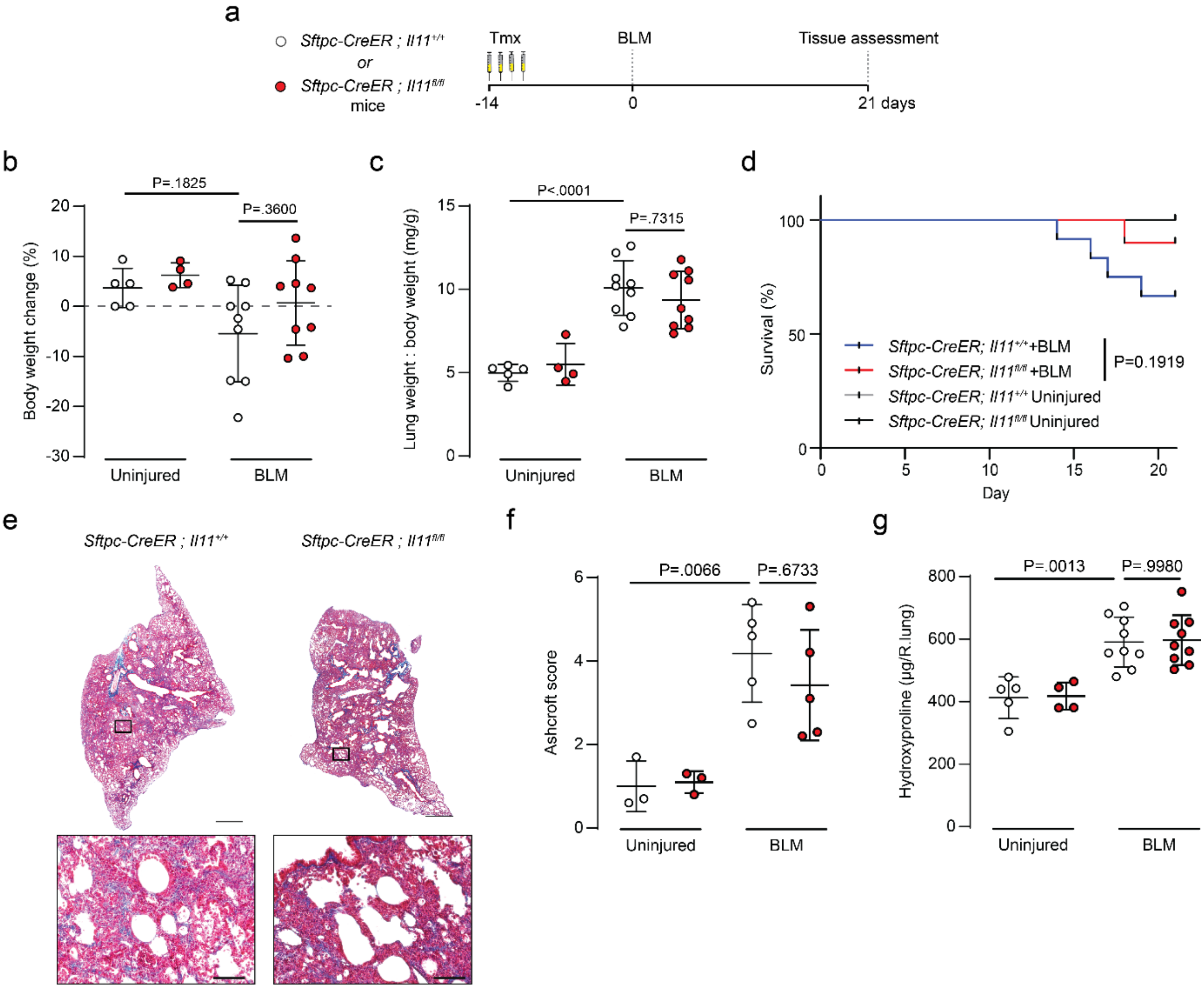

Supplementary Figure 18. Mice with AT2 cell specific *Il11* deletion are not protected against bleomycin-induced lung fibrosis. (**a**) Schematic showing the period of tamoxifen (Tmx) administration and the induction of lung fibrosis in *Sftpc-CreER; Il11$^{fl/fl}$ or Il11$^{+/+}$* mice via oropharyngeal injection of bleomycin (BLM). (**b**) Percentage body weight change post-BLM injury (day 21 versus day 0), (**c**) lung weight to body weight indices and (**d**) survival analysis of tamoxifen-treated *Sftpc-CreER; Il11$^{fl/fl}$ or Il11$^{+/+}$* mice 21 days post-BLM injury. (**e**) Representative images of Masson's trichrome staining, (**f**) lung histopathological fibrosis scoring and (**g**) lung hydroxyproline content in the right lungs of tamoxifen-treated *Sftpc-CreER; Il11$^{fl/fl}$ or Il11$^{+/+}$* mice 21 days post-BLM injury. Scale bars in **e**: 2000 µm (top panels) 100 µm (bottom panels). Data are represented as mean ± s.d. *P* values were determined by one-way ANOVA (Tukey's test) in panel **b**, **c**, **f**, **g** and by Log-rank (Mantel-Cox) test in panel **d**; n = 3 control and 9 BLM-treated mice / genotype.

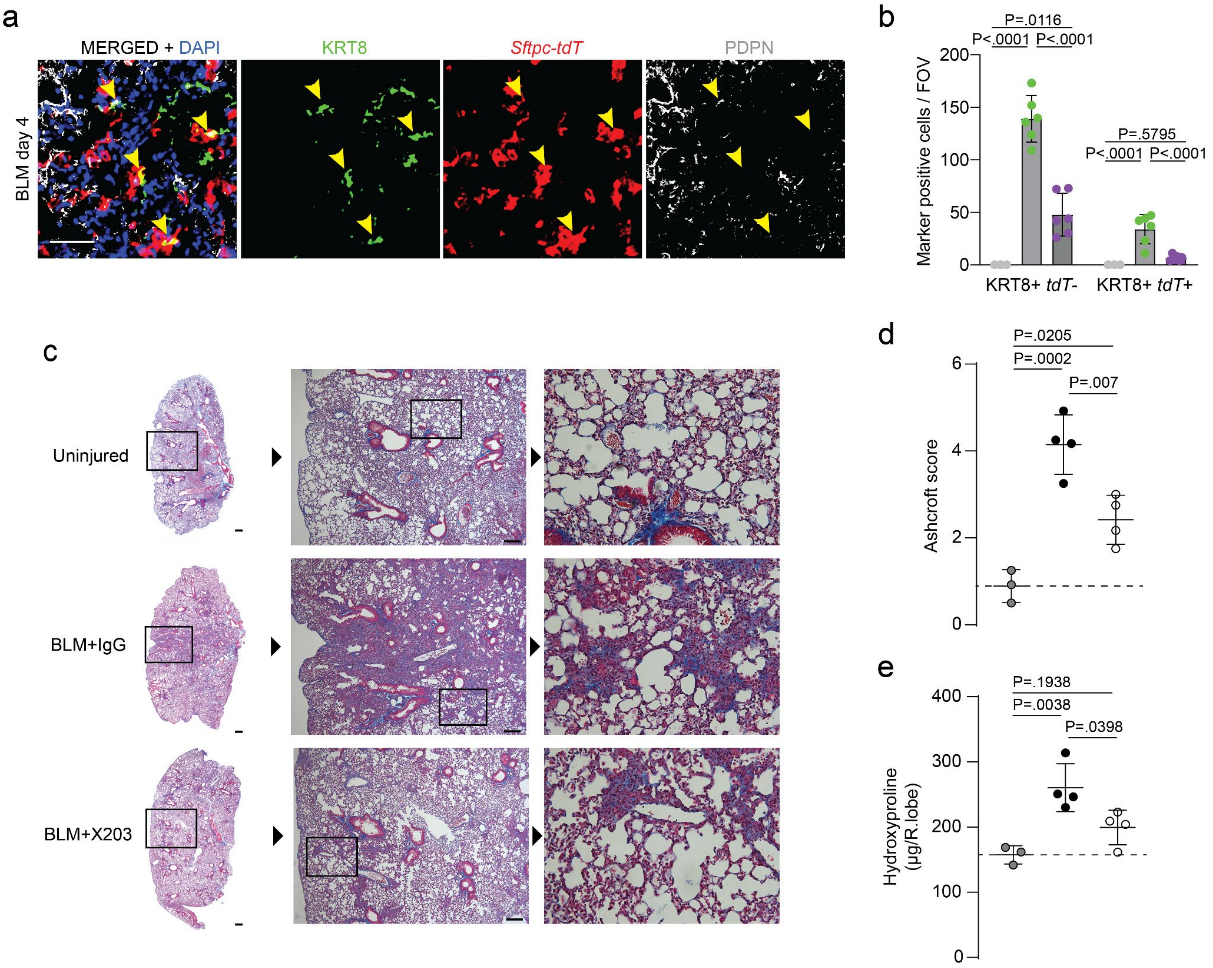

Supplementary Figure 19. Pharmacological inhibition of IL11 reduces the accumulation of alveolar epithelial transitional cells and attenuates bleomycin-induced lung fibrosis. (a) Representative images of immunostaining for KRT8 and PDPN in the lung of *Sftpc-tdT* mice 4 days post-BLM challenge. (**b**) Quantification of KRT8^{+} cells in *tdT*$^{+}$ or t*dT*$^{-}$ cells from the lungs of *Sftpc-tdT* mice treated with either IgG or X203 antibodies 12 days post-BLM-injury. n = 3 control and 6 BLM-treated mice / group. (**c**) Representative images of Masson's trichrome staining, (**d**) lung histopathological fibrosis scoring and (**e**) lung hydroxyproline content of right caudal lung lobes from *Sftpc-tdT* mice treated with either IgG or X203 antibodies 12 days post-BLM-injury. Scale bars in **a**: 100 µm; **c**: 1000 µm (left panel) and 100 µm (center panel). Data are representative of at least three independent experiments and quantification data are shown as mean ± s.d. Dotted lines represent mean values of uninjured controls. *P* values were determined by one-way ANOVA (Tukey's test).

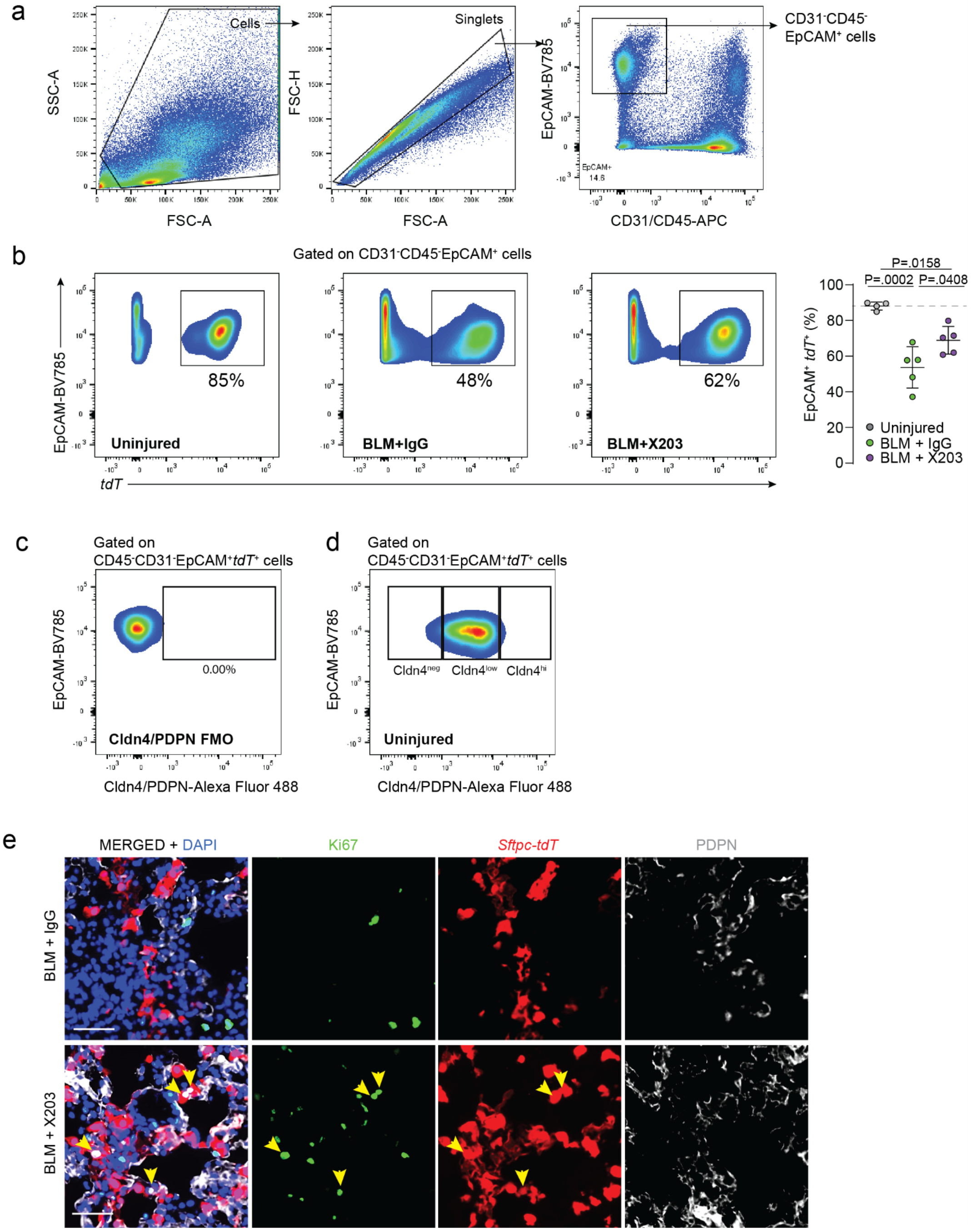

Supplementary Figure 20. Pharmacological inhibition of IL11 promotes AT2 cell proliferation after bleomycin-induced lung injury. (**a-b**) Flow cytometry gating and quantification of CD31$^-$ CD45$^-$ EpCAM$^+$ *tdT*$^+$ lung cells from *Sftpc-tdT* mice treated with X203 or IgG antibodies 12 days post-BLM injury. *P* values were determined by one-way ANOVA (Tukey's test). n = 4 controls and 5 BLM-treated mice / group. (**c**) FMO control of Cldn4- or PDPN-Alexa Fluor 488 and (**d**) gating strategy for Cldn4 subsets in CD31$^-$ CD45$^-$ EpCAM$^+$ *tdT*$^+$ lung cells from *Sftpc-tdT* mice treated with X203 or IgG antibodies 12 days post-BLM injury. (**e**) Representative images of immunostaining for Ki67 and PDPN in the lung from *Sftpc-tdT* mice treated with X203 or IgG antibodies 12 days post-BLM injury. Data are representative of at least three independent experiments. Scale bars: 50 µm.

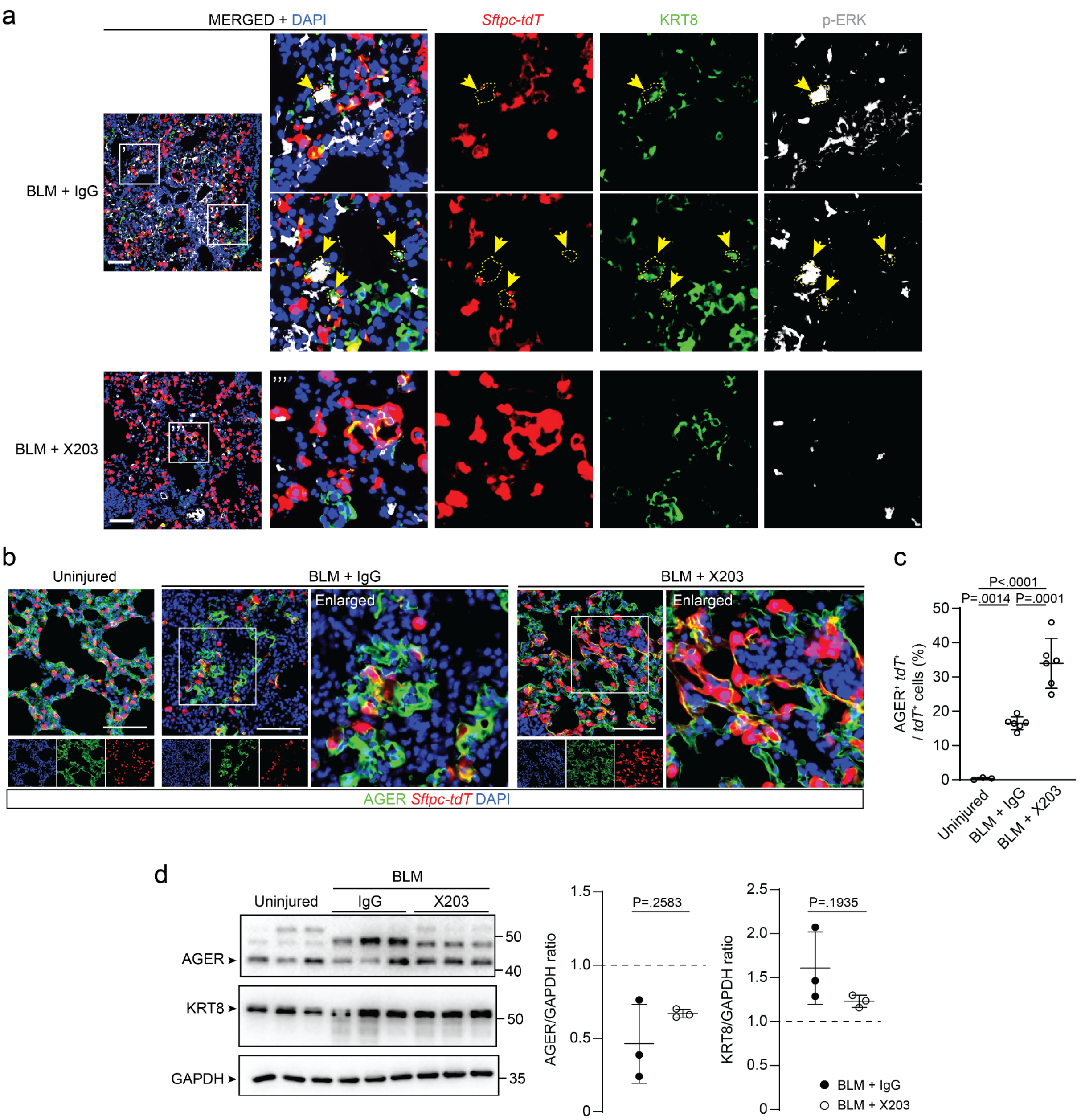

Supplementary Figure 21. Pharmacological inhibition of IL11 reduces p-ERK$^+$ KRT8$^+$ transitional epithelial cells after bleomycin-induced lung injury. (**a**) Representative images of immunostaining for Ki67 and PDPN or (**b**) KRT8 and p-ERK in the lung from *Sftpc-CreER; R26-tdT* mice treated with X203 or IgG antibodies (12 days post-BLM). Scale bars: 50 µm in **a** and 100 µm in **b**. Yellow arrowheads indicate Ki67$^+$ *tdT*$^+$ cells in panel **a** or p-ERK$^+$ KRT8$^+$ *tdT*$^+$ cells in panel **b**. (**c**) Representative images of immunostaining for AGER and (**d**) the ratio of AGER$^+$ *tdT*$^+$ to *tdT*$^+$ cells in lung sections from BLM-injured *Sftpc-tdT* mice treated with X203 or IgG antibodies. Scale bars: 100 µm. n = 3 control and 6 BLM-treated mice / group. Data in **a** and **b** are representative of three independent experiments. (**d**) Western blot and densitometry analysis of mature AGER and KRT8 expression in lung homogenates from *Sftpc-tdT* mice treated with either X203 or IgG antibodies 12 days post-BLM injury. n = 3 mice / group. KRT8 (~55 kDa band) and AGER protein (~43 kDa band) were quantified by densitometry. GAPDH control was performed on different membranes. Uncropped blots are shown in Supplementary Figure 24. Data are represented as mean ± s.d. *P* values were determined by one way ANOVA (Tukey's test) in **c** or by two tailed Student's *t*-test in panel in **d**.

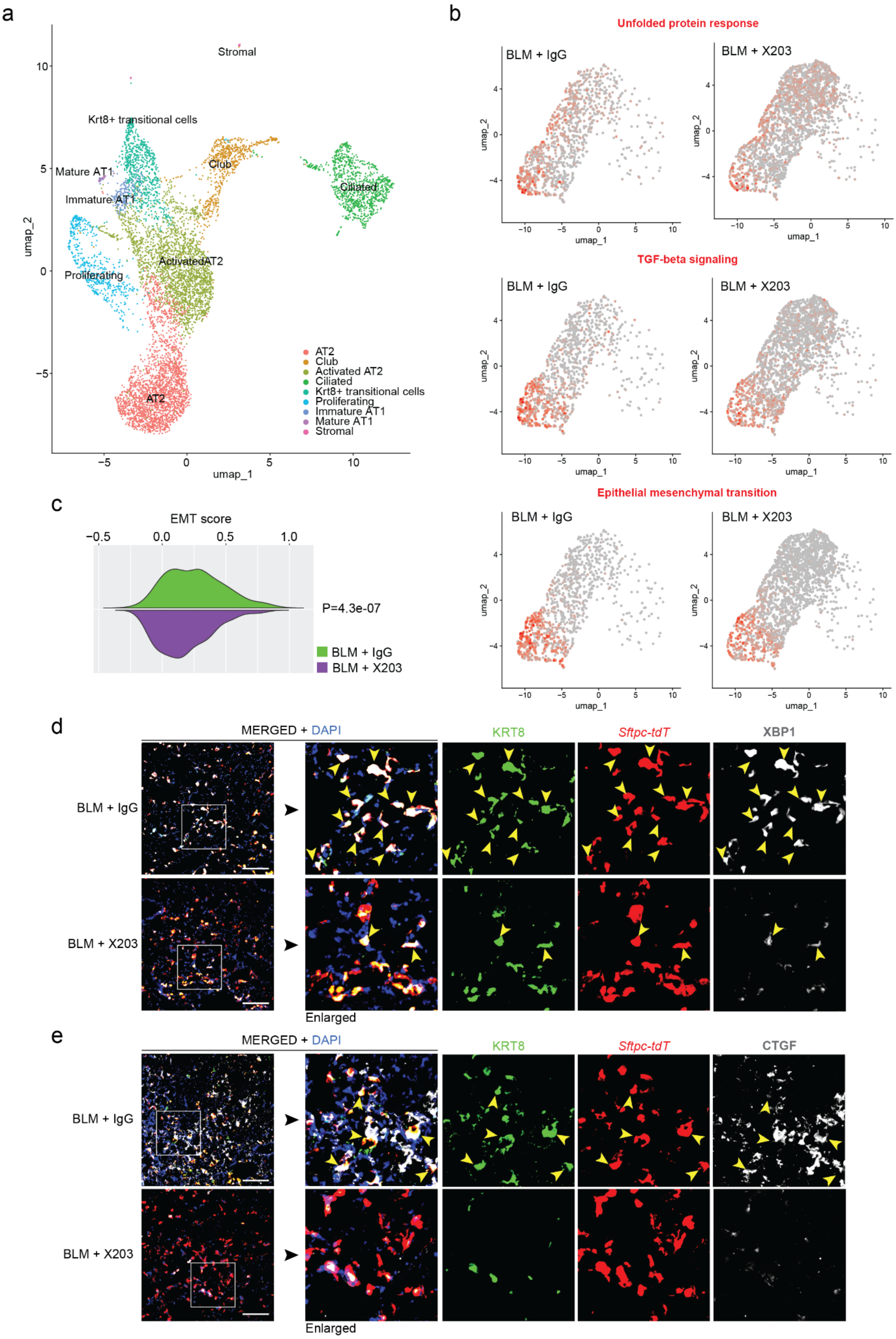
a
Stromal
Krt8+ transitional cells
Mature AT1
Immature AT1
Club
Ciliated
ActivatedAT2
Proliferating
AT2
AT2
Club
Activated AT2
Ciliated
Krt8+ transitional cells
Proliferating
Immature AT1
Mature AT1
Stromal
umap_1
umap_2
b
Unfolded protein response
TGF-beta signaling
Epithelial mesenchymal transition
BLM + IgG
BLM + X203
c
EMT score
P=4.3e-07
BLM + IgG
BLM + X203
d
MERGED + DAPI
KRT8
Sftpc-tdT
XBP1
BLM + IgG
BLM + X203
Enlarged
e
MERGED + DAPI
KRT8
Sftpc-tdT
CTGF
BLM + IgG
BLM + X203
Enlarged

Supplementary Figure 22. Pharmacological inhibition of IL11 attenuates profibrotic signatures of transitional epithelial cells after bleomycin-induced lung injury. (**a**) UMAP visualization of lung epithelial cells colored by cell identity (K = 7692 cells). (**b**) UMAP visualization single cells colored by gene expression signature scores for the indicated pathways from the MSigDB Hallmark gene sets AT2-lineage cells from X203 or IgG treated mice post-BLM-injury. Respective cell clusters are shown in main Fig. 5g. (**c**) Violin plot visualization of EMT score based on leading edge analysis of EMT Hallmark (GSEA) genes in Krt8+ transitional cells. (**d-e**) Representative images of immunostaining of KRT8 and (**d**) XBP1 or (**e**) CTGF in the lungs of X203 or IgG treated mice post-BLM-injury. *P* value in **c** was determined by Wilcoxon signed-rank test. Data in **d** and **e** are representative of three independent experiments.

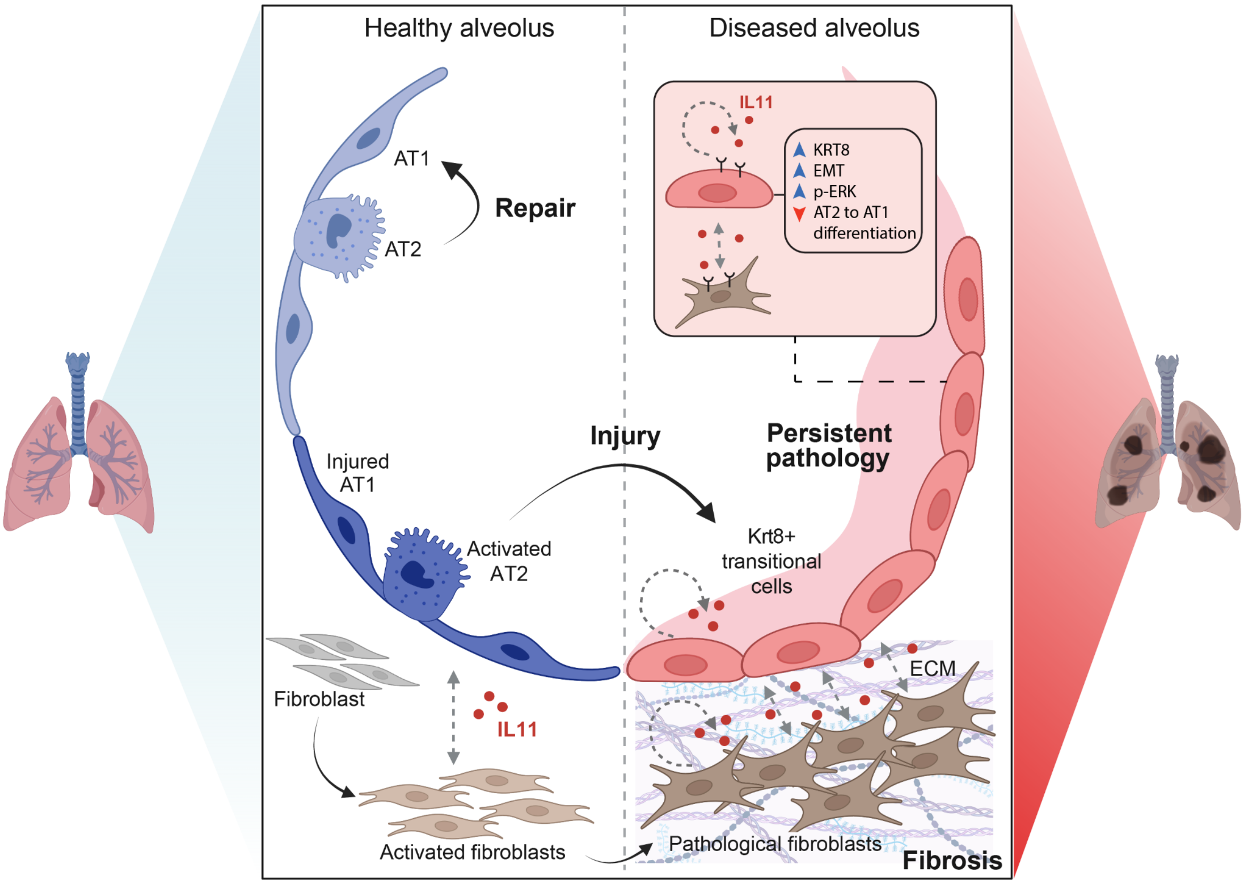

Supplementary Figure 23. Schematic illustrating the proposed effects of IL11 in the healthy or fibrotic alveolar niche. During alveolar regeneration, AT2 cells differentiate into AT1 cells via a Krt8+ transitional cell state. However, in the context of persistent/severe pathology, aberrant ECM-producing Krt8+ transitional cells with defective AT1 differentiation capacity accumulate in the lung and the factors that regulate the differentiation and maintenance of these aberrant cells are poorly understood. In the diseased lung, IL11 is upregulated by activated AT2 cells, Krt8+ transitional cells and pathological fibroblasts. In AT2 cells, IL11 stimulates ERK signaling pathway dependent pro-EMT programs that maintain AT2 cells in a dysfunctional $KRT8^{+}$ state with impaired alveolar epithelial regeneration capacity. IL11 induces the expression of pathologic ECM proteins (such as Collagen I and CTGF) by $KRT8^{+}$ cells which may directly promote aberrant lung remodeling. Additionally, the elevated expression of IL11 by pathological fibroblasts in the fibrotic niche may contribute to aberrant epithelial cell differentiation in lung fibrosis. Created in BioRender. Lim, W. (2023) BioRender.com/u32j895

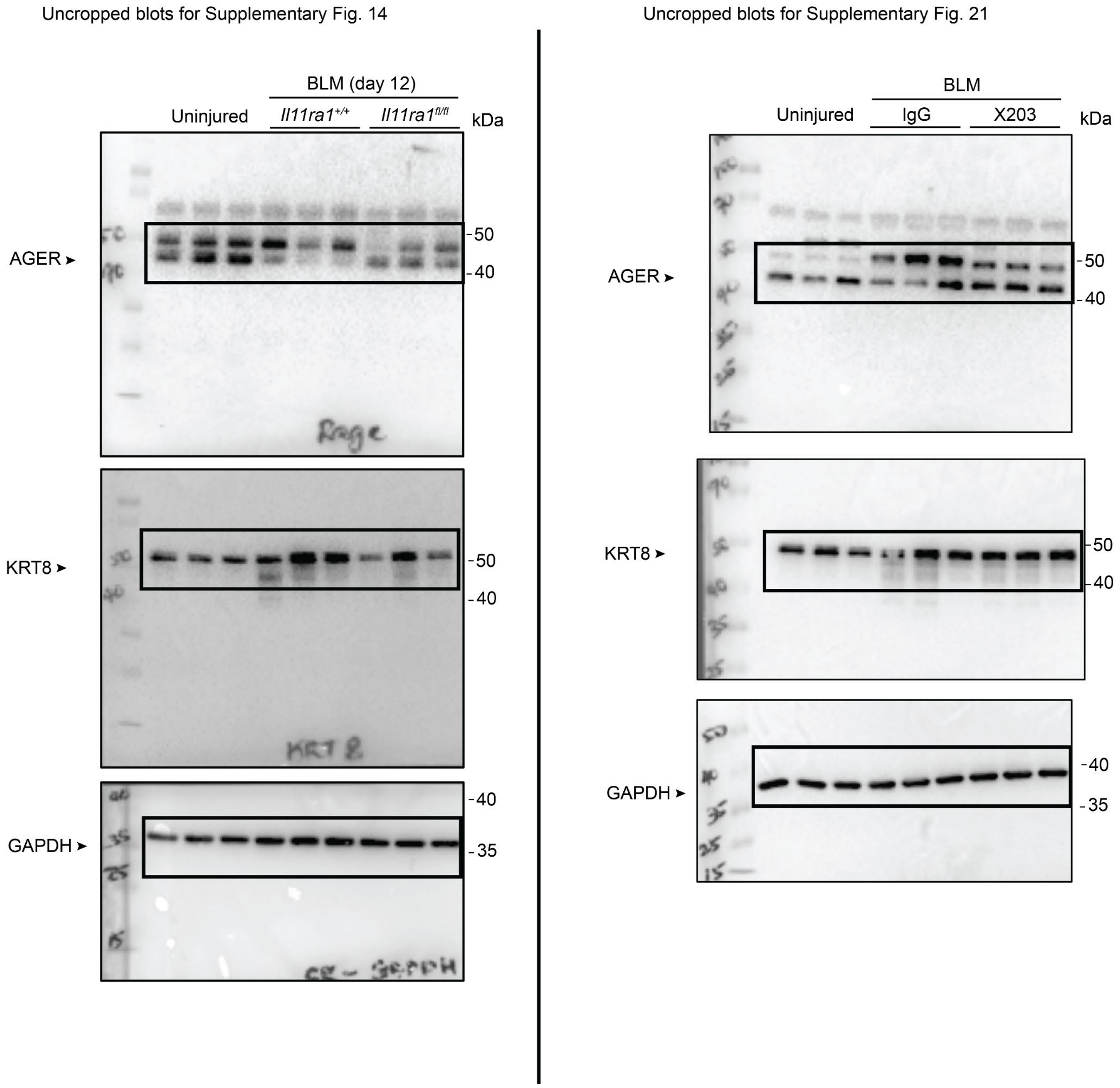

Supplementary Figure 24. Uncropped blots.
