# Supplementary material for: Interleukin-11 causes alveolar type 2 cell dysfunction and prevents alveolar regeneration
Source: Nat Commun. 2024 Oct 2;15:8530. doi: 10.1038/s41467-024-52810-8 (PMC11448503; doi:10.1038/s41467-024-52810-8)
Supplement: Supplementary file 3 — Description of Additional Supplementary Files [file 41467_2024_52810_MOESM3_ESM.pdf]

Description for Supplementary Data files

File Name: Supplementary Data 1
Description: This file contains the gene enrichment analysis of IL11, IL11RA and IL6 in human lung scRNA-seq datasets.

File Name: Supplementary Data 2
Description: This file contains the pathway analysis of IL11 co-expressed genes in human lung scRNA-seq datasets.

File Name: Supplementary Data 3
Description: This file contains the genes co-expressed with IL11 in human lung scRNA-seq datasets.

File Name: Supplementary Data 4
Description: This file contains the top 100 significantly upregulated genes in TGFβ1-stimulated HPAEpiC compared to baseline.

File Name: Supplementary Data 5
Description: This file contains the enriched pathways in KRT8+ transitional cells from bleomycin-injured Sftpc-CreER; Il11ra1-floxed vs. control mice.

File Name: Supplementary Data 6
Description: This file contains the differentially expressed genes in Krt8+ transitional cells between bleomycin-injured Sftpc-CreER; Il11ra1-floxed vs. control mice.

File Name: Supplementary Data 7
Description: This file contains the enriched pathways in Krt8+ transitional cells from bleomycin-injured Sftpc-tdT mice treated with X203 vs. IgG antibodies.
