# Supplementary material for: Interleukin-11 causes alveolar type 2 cell dysfunction and prevents alveolar regeneration
Source: Nat Commun. 2024 Oct 2;15:8530. doi: 10.1038/s41467-024-52810-8 (PMC11448503; doi:10.1038/s41467-024-52810-8)
Supplement: Supplementary file 11 — Reporting Summary [file 41467_2024_52810_MOESM11_ESM.pdf]

Corresponding author(s): Benjamin Ng, Stuart Cook

Last updated by author(s): Aug 22, 2024

Reporting Summary

Nature Portfolio wishes to improve the reproducibility of the work that we publish. This form provides structure for consistency and transparency in reporting. For further information on Nature Portfolio policies, see our Editorial Policies and the Editorial Policy Checklist.

Statistics

For all statistical analyses, confirm that the following items are present in the figure legend, table legend, main text, or Methods section.

n/a	Confirmed	
☐	☒	The exact sample size (*n*) for each experimental group/condition, given as a discrete number and unit of measurement
☐	☒	A statement on whether measurements were taken from distinct samples or whether the same sample was measured repeatedly
☐	☒	The statistical test(s) used AND whether they are one- or two-sided<br>*Only common tests should be described solely by name; describe more complex techniques in the Methods section.*
☒	☐	A description of all covariates tested
☐	☒	A description of any assumptions or corrections, such as tests of normality and adjustment for multiple comparisons
☐	☒	A full description of the statistical parameters including central tendency (e.g. means) or other basic estimates (e.g. regression coefficient) AND variation (e.g. standard deviation) or associated estimates of uncertainty (e.g. confidence intervals)
☐	☒	For null hypothesis testing, the test statistic (e.g. F, t, r) with confidence intervals, effect sizes, degrees of freedom and P value noted<br>*Give P values as exact values whenever suitable.*
☒	☐	For Bayesian analysis, information on the choice of priors and Markov chain Monte Carlo settings
☒	☐	For hierarchical and complex designs, identification of the appropriate level for tests and full reporting of outcomes
☒	☐	Estimates of effect sizes (e.g. Cohen's d, Pearson's r), indicating how they were calculated

Our web collection on statistics for biologists contains articles on many of the points above.

Software and code

Policy information about availability of computer code

Data collection	Fluorescence intensity of immunostaining was collected and analysed on the Harmony software version 3.5.2 (PerkinElmer) and Columbus software (version 2.7.2, PerkinElmer) or on the LAS X software (version 3.0.1, Leica). FACS data was collected using FACSDiva (version 8.0.1 BD Biosciences)
Data analysis	Single-cell analysis for human lung cells were ran in R environment (R version 4.0.5) with the following packages, slingshot (v 1.8.0), princurve (v 2.1.6), igraph (v 1.3.5), cowplot (v 1.1.1), qvalue (v 2.22.0), stringr (v 1.5.0), ggplot2 (v 3.3.6), Matrix (v 1.5-1), data.table (v 1.14.4), SeuratObject (v 4.1.2), enrichR (v 3.1), and Seurat (v 4.2.0). IL11 co-expression network was visualized in Cytoscape (v 3.8.2).<br>Single cell analysis on mouse cells were ran in R environment (R version 4.2.0) with the following packages, Biobase (v2.58.0), BiocGenerics (v0.44.0), BiocParallel (v1.32.6), data.table (v1.14.8), DESeq2 (v1.38.3), dplyr (v1.1.2), fgsea (v1.24.0), GenomeInfoDb (v1.34.9), GenomicRanges (v1.50.2), ggplot2 (v3.4.2), IRanges (v2.32.0), magrittr (v2.0.3), Matrix (v1.6-5), Matrix.utils (v0.9.8), MatrixGenerics (v1.10.0), matrixStats (v1.0.0), msigdbr (v7.5.1), pheatmap (v1.0.12), princurve (v2.1.6), purrr (v1.0.1), RColorBrewer (v1.1-3), reshape (v0.8.9), S4Vectors (v0.36.2), scCustomize (v2.0.1), Seurat (v5.0.1), SeuratObject (v5.0.1), SingleCellExperiment (v1.20.1), slingshot (v2.6.0), sp (v2.0-0), stringr (v1.5.0), SummarizedExperiment (v1.28.0), tibble (v3.2.1), TrajectoryUtils (v1.6.0).<br><br>Immunofluorescence image quantification were analysed using Harmony software (v3.5.2, PerkinElmer), Columbus software (v2.7.2, PerkinElmer) and Fiji software (v1.45). FACS data were anlysed using FlowJo (v10). Statistical analysis was performed using Graphpad Prism (v9.4.1) software.

For manuscripts utilizing custom algorithms or software that are central to the research but not yet described in published literature, software must be made available to editors and reviewers. We strongly encourage code deposition in a community repository (e.g. GitHub). See the Nature Portfolio guidelines for submitting code & software for further information.

Data

Policy information about availability of data

All manuscripts must include a data availability statement. This statement should provide the following information, where applicable:

- Accession codes, unique identifiers, or web links for publicly available datasets
- A description of any restrictions on data availability
- For clinical datasets or third party data, please ensure that the statement adheres to our policy

All data associated with this study are presented in the paper or in the Supplementary Materials. Sequencing data are has been uploaded onto Gene Expression Omnibus under the accession GSE261794. Source data are provided with this paper. All codes generated for this study are available on Zenodo repository [doi.org/10.5281/zenodo.13315637] and GitHub link: [https://github.com/henryhuang12345/IL11_AEC_2024].

Research involving human participants, their data, or biological material

Policy information about studies with human participants or human data. See also policy information about sex, gender (identity/presentation), and sexual orientation and race, ethnicity and racism.

Reporting on sex and gender	Not relevant to this study.
Reporting on race, ethnicity, or other socially relevant groupings	*Please specify the socially constructed or socially relevant categorization variable(s) used in your manuscript and explain why they were used. Please note that such variables should not be used as proxies for other socially constructed/relevant variables (for example, race or ethnicity should not be used as a proxy for socioeconomic status).*<br>*Provide clear definitions of the relevant terms used, how they were provided (by the participants/respondents, the researchers, or third parties), and the method(s) used to classify people into the different categories (e.g. self-report, census or administrative data, social media data, etc.)*<br>*Please provide details about how you controlled for confounding variables in your analyses.*
Population characteristics	*Describe the covariate-relevant population characteristics of the human research participants (e.g. age, genotypic information, past and current diagnosis and treatment categories). If you filled out the behavioural & social sciences study design questions and have nothing to add here, write "See above."*
Recruitment	*Describe how participants were recruited. Outline any potential self-selection bias or other biases that may be present and how these are likely to impact results.*
Ethics oversight	*Identify the organization(s) that approved the study protocol.*

Note that full information on the approval of the study protocol must also be provided in the manuscript.

Field-specific reporting

Please select the one below that is the best fit for your research. If you are not sure, read the appropriate sections before making your selection.

[x] Life sciences [ ] Behavioural & social sciences [ ] Ecological, evolutionary & environmental sciences

For a reference copy of the document with all sections, see nature.com/documents/nr-reporting-summary-flat.pdf

Life sciences study design

All studies must disclose on these points even when the disclosure is negative.

Sample size	Sample sizes for in vivo studies were based on the authors experience with the models and from previous publications (PMID:31554736; PMID:32656894; PMID:34239012). In general, in vivo experiments were designed to detect 20% differences (80% power; α= 0.05) between treatment / genotype-dependent groups. Sample sizes for scRNA-seq and in vitro assays were determined based on prior studies in the field and varied based on animal availability. In vitro studies were performed in duplicates on at least 2 biological replicates. scRNA-seq experiments on enriched lung epithelial cells from Sftpc-CreER;Il11ra1-floxed mice (6 mice, K=36443 cells). scRNA-seq experiments enriched lung epithelial cells from Sftpc-CreER;R26-tdTomato mice treated with X203/IgG antibodies (3 mice, K=7692 cells).
Data exclusions	Outliers for in vitro and in vivo data were determined by ROUTS test (Q=1%) and excluded from analysis. For scRNA-seq data, cells were filtered for quality control purposes. No other data were excluded from analysis.
Replication	In vitro experiments were performed in duplicates and consistent results were obtained on all replication attempts. In vitro experiments were conducted on at least 2 biological replicates and in vivo experiments were performed on at least 3 biological replicates per group and

	confirmed with successful replication.
Randomization	Randomization was not applied to the various transgenic mice used in the study as they were assigned to treatment groups (Bleomycin or vehicle) based on their genotypes. For Bleomycin studies involving anti-IL11 treatment, mice were randomly assigned to experimental groups on the day prior to treatment. No randomization was conducted for in vitro studies.
Blinding	Histopathological assessment of lung fibrosis were performed blinded to genotypes. Investigators were not blinded to group allocations in other analyses.

Reporting for specific materials, systems and methods

We require information from authors about some types of materials, experimental systems and methods used in many studies. Here, indicate whether each material, system or method listed is relevant to your study. If you are not sure if a list item applies to your research, read the appropriate section before selecting a response.

Materials & experimental systems

n/a	Involved in the study
☐	☒ Antibodies
☐	☒ Eukaryotic cell lines
☒	☐ Palaeontology and archaeology
☐	☒ Animals and other organisms
☒	☐ Clinical data
☒	☐ Dual use research of concern
☒	☐ Plants

Methods

n/a	Involved in the study
☒	☐ ChIP-seq
☐	☒ Flow cytometry
☒	☐ MRI-based neuroimaging

Antibodies

Antibodies used	Primary antibodies used include: anti-KRT8 (Merck Millipore, clone TROMA-1, MABT329), anti-p-ERK (Cell Signaling Technology, clone D13.14.4E, 4370 or clone D1H6G, 5726; 1:100 dilution), anti-Ki67 (Abcam, ab16667; 1:100 dilution), anti-SFTPC (Abcam, clone EPR19839, ab211326; 1:100 dilution), anti-SP-C (Santa Cruz Biotech., clone H-8, c-518029; 1:200 dilution), anti-GFP (Abcam, ab290 / ab6673; 1:200 dilution), anti-AGER (R&D Systems, clone # 175410, MAB1179; 1:1-200 dilution), anti-Podoplanin (R&D Systems, AF3244; 1:200 dilution), anti-IL-11 (Invitrogen, PAS-95982; 1:100 dilution), anti-CD45 (Proteintech, 20103-1-AP; 1:50 dilution), anti-PDGFRA (R&D Systems, AF1062; 1:100 dilution), anti-Collagen I (Abcam, ab21286, ab34710; 1:100 dilution), anti-CTGF (Abcam, ab6992; 1:100 dilution), anti-XBP1 (Abcam, ab37152; 1:100 dilution), anti-fibronectin (Abcam, ab2413; 1:100 dilution), anti-IL-11RA (Abcam, clone EPR5446, ab125015; 1:100 dilution).<br>EpCAM-BV785 (BioLegend #118245; 1:200 dilution), CD45-APC (BioLegend, 103112; 1:200 dilution), CD31-APC/Cy7 (BioLegend, 102534; 1:200 dilution), I-A/I-E - AlexaFluor488 (MHC-II) (BioLegend, 107616; 1:200 dilution), anti-Claudin 4 (Invitrogen, clone ZMD.306, 36-4800; 1:100 dilution) and PE-conjugated anti-GFP (Abcam, clone EPR14104, ab303588; 1:100 dilution).<br>Secondary antibodies used are as follows: Donkey anti-Rabbit/Goat Alexa Fluor Plus 555 (Life Technologies; A-31572 / A-32816), Donkey anti-Mouse/Rabbit/Goat Alexa Fluor Plus 647 (Life Technologies; A-32787 / A32795 / A32849), Donkey anti-Mouse/Rabbit/ Goat Alexa Fluor Plus 488 (A32766 / AA32790 / A32814). Custom-made primary antibodies used in in vitro / in vivo neutraization studies: Monoclonal anti-IL11 (X203), IgG (11E10) were custom made by Genovac.
Validation	The primary and secondary antibodies used in this study were purchased from and validated by the various commercial vendors and have been cited in several peer reviewed publications. The development and validation of X203 was described in our previous study (PMID:31554736).<br><br>Manufacturer's website containing validation data are listed below:<br>1. anti-KRT8 (https://www.merckmillipore.com/SG/en/product/Anti-Cytokeratin-8-Antibody-clone-TROMA-1,MM_NF-MABT329?ReferrerURL=https%3A%2F%2Fwww.google.com%2F)<br>2. anti-p-ERK (https://www.cellsignal.com/products/primary-antibodies/phospho-p44-42-mapk-erk1-tyr204-erk2-tyr187-d1h6g-mouse-mab/5726)<br>3. anti-p-ERK (https://www.cellsignal.com/products/primary-antibodies/phospho-p44-42-mapk-erk1-2-thr202-tyr204-d13-14-4e-xp-rabbit-mab/4370?srsltid=AfmBOoqQ-REoy_BkLmmwWlk9Z5PsaP4uw4zd2XgifMcGi5bjpl2pQOhR)<br>4. anti-Ki67 (https://www.abcam.com/en-us/products/primary-antibodies/ki67-antibody-sp6-ab16667)<br>5. anti-SFTPC (https://www.abcam.com/en-us/products/primary-antibodies/prosurfactant-protein-c-antibody-epr19839-ab211326)<br>6. anti-SP-C (https://www.scbt.com/p/sp-c-antibody-h-8?gad_source=1&gclid=Cj0KCQjww5u2BhDeARIsALBuLnNHkx7qAi2_C4q1uRNjvuWjRUmh8FTMrtghlt2Lhh4GaV2oWHGvtoEaAprhEALw_wcB)<br>7. anti-GFP (https://www.abcam.com/en-us/products/primary-antibodies/gfp-antibody-ab290)<br>8. anti-GFP (https://www.abcam.com/en-us/products/primary-antibodies/gfp-antibody-ab6673)<br>9.anti-RAGE (https://www.rndsystems.com/products/mouse-rat-rage-antibody-175410_mab1179)<br>10. anti-Podoplanin (https://www.rndsystems.com/products/mouse-podoplanin-antibody_af3244)<br>11. anti-IL11 (https://www.thermofisher.com/antibody/product/IL-11-Antibody-Polyclonal/PA5-95982)<br>12. anti-CD45 (https://www.ptglab.com/products/RPTPC-Antibody-20103-1-AP.htm)<br>13. anti-PDGFRA (https://www.rndsystems.com/products/mouse-pdgf-ralpha-antibody_af1062)<br>14. anti-Collagen I (https://www.abcam.com/en-us/products/primary-antibodies/collagen-i-antibody-ab34710#)

15. anti-Collagen I (https://www.abcam.com/en-us/products/primary-antibodies/collagen-i-antibody-ab21286)
16. anti-CTGF (https://www.abcam.com/en-us/products/primary-antibodies/ctgf-antibody-ab6992#)
17. anti-XBP1 (https://www.abcam.com/en-us/products/primary-antibodies/xbp1-antibody-ab37152#)
18. anti-Fibronectin (https://www.abcam.com/en-us/products/primary-antibodies/fibronectin-antibody-ab2413)
19. anti-IL-11RA (https://www.abcam.com/en-us/products/primary-antibodies/il-11ra-antibody-epr5446-ab125015)
20. BV785 anti mouse EpCAM (https://punchout.biolegend.com/en-us/products/brilliant-violet-785-anti-mouse-cd326-ep-cam-antibody-19458)
21. APC anti-mouse CD45 (https://punchout.biolegend.com/en-us/products/apc-anti-mouse-cd45-antibody-97)
22. APC/Cy7 anti-mouse CD31 (https://punchout.biolegend.com/en-us/products/apccyanine7-anti-mouse-cd31-antibody-19419)
23. Alexa Fluor 488 anti-mouse I-A/I-E (https://punchout.biolegend.com/en-us/products/alexa-fluor-488-anti-mouse-i-a-i-e-antibody-3134)
24. anti-Claudin 4 (https://www.thermofisher.com/antibody/product/Claudin-4-Antibody-clone-ZMD-306-Polyclonal/36-4800)
25. PE Anti-GFP (https://www.abcam.com/en-bw/products/primary-antibodies/pe-gfp-antibody-epr14104-ab303588)

Eukaryotic cell lines

Policy information about cell lines and Sex and Gender in Research

Cell line source(s)	Human pulmonary alveolar epithelial cells (HPAEpiC) (ScienCell Research Laboratories, 3200),<br>Human small airway epithelial cells (HSAEC) (Lonza Bioscience, CC-2547).
Authentication	The cells were purchased from and authenticated by the commercial vendors (ScienCell and Lonza Bioscience).
Mycoplasma contamination	All cells were tested negative for mycoplasma.
Commonly misidentified lines<br>(See ICLAC register)	No commonly misidentified lines were used.

Animals and other research organisms

Policy information about studies involving animals; ARRIVE guidelines recommended for reporting animal research, and Sex and Gender in Research

Laboratory animals	Animals were maintained in a specific pathogen-free environment and had ad libitum access to food and water, with a 12 hour light/dark cycle, at an ambient temperature of 21-24°C and humidity of 40-70%. The following mice strains were maintained on C57BL/6 background and used for the study:<br>1. Sftpctm1(cre/ERT2)Blh (Sftpc-CreER)<br>2. B6.Cg-Gt(ROSA)26Sortm9(CAG-tdTomato)Hze/J (R26-tdTomato mice)<br>3. C57BL/6-Il11ra1em1Cook/J (Il11ra1 floxed mice)<br>4. C57BL/6-Il11tm1.1Cook/J (IL11EGFP reporter mice)<br>5.Il11 floxed mice (PMID:35806094).<br>6. SftpcCreER mice were crossed with R26-tdTomato mice to generate Sftpc-tdT mice.<br>7. Sftpc-CreER mice were crossed with either Il11ra1 floxed or Il11 floxed mice to generate Sftpc-tdt; Il11ra1-floxed mice.<br>8. Sftpc-CreER; Il11ra1-floxed mice were further crossed with Sftpc-tdT mice to generate Sftpc-tdt; Il11ra1-floxed mice.
Wild animals	No wild animals were used in this study.
Reporting on sex	Male mice were used in this study.
Field-collected samples	No field collected samples were obtained in this study.
Ethics oversight	All animal procedures were approved and performed in accordance with guidelines set by the Institutional Animal Care and Use Committee at SingHealth (Singapore) and the SingHealth Institutional Biosafety Committee and with the recommendations in the (ARRIVE) guidelines.

Note that full information on the approval of the study protocol must also be provided in the manuscript.

Plants

Seed stocks: Not applicable to the study.

Novel plant genotypes: Not applicable to the study.

Authentication: Not applicable to the study.

Flow Cytometry

Plots

Confirm that:

- [x] The axis labels state the marker and fluorochrome used (e.g. CD4-FITC).
- [x] The axis scales are clearly visible. Include numbers along axes only for bottom left plot of group (a 'group' is an analysis of identical markers).
- [x] All plots are contour plots with outliers or pseudocolor plots.
- [x] A numerical value for number of cells or percentage (with statistics) is provided.

Methodology

Sample preparation: Mouse lungs were perfused with cold sterile saline through the right ventricle. The lungs were then intratracheally inflated with 1.5 ml of Dispase 50 U/ml (Corning) followed by installation of 0.5 ml of 1% low melting agarose (BioRad) via the trachea. The lungs were excised and incubated on an orbital shaker for 45 min at room temperature. Each lobe was then minced into small pieces in DMEM (GIBCO) supplemented with 10% FBS (GIBCO) and 0.33 U/ml DNase I (Roche) and placed on the orbital shaker for an additional 10 minutes. The cells were then filtered through a 100 µm cell strainer and centrifuged at 400 g for 5 min at 4°C. The cell pellet was resuspended in ACK-buffer (GIBCO), incubated for 2 min on ice and then filtered through a 40 µm cell strainer. The cells were centrifuged at 400 g for 5 min at 4°C and resuspended in DPBS (GIBCO) supplemented with 5% FBS, and then stained with the primary antibodies.

Instrument: BD FACS Aria III system (BD Bioscience), BD LSR Fortessa system (BD Biosciences).

Software: FACSDiva (v8.0.1 BD Biosciences)

Cell population abundance: Enriched populations of mouse lung epithelial cells of up to 20% of the live cell fraction were obtained from FACS and used for scRNA-seq experiments.

Gating strategy: We firstly applied FSC and SSC gating to exclude debris and doublets, followed by live/dead cell determination by DAPI staining. Epithelial cells were gated on EpCAM+ CD31- CD45- cells. Transitional epithelial cells in Sftpc-tdT mice were gated by CD31- CD45- EpCAM+ tdT+ Cldn4hi cells. Transitional AT1 cells were gated by EpCAM+ CD31- CD45- EpCAM+ tdT+ PDPN+ cells. Detailed plots of cell gating strategies are presented in the supplementary figures.

- [x] Tick this box to confirm that a figure exemplifying the gating strategy is provided in the Supplementary Information.
